# How emotional response styles of generative AI companions shape adaptive emotion regulation and short-term AI reliance

**DOI:** 10.3389/fpsyg.2026.1950163

**Published:** 2026-09-08

**Authors:** Chun Wang

**Affiliations:** School of Art and Design, Guangzhou College of Commerce, Guangzhou, Guangdong, China

**Keywords:** AI companions, emotion regulation, emotional response style, emotion-regulation self-efficacy, generative artificial intelligence, perceived AI necessity, short-term AI reliance

## Abstract

**Introduction:**

Generative artificial intelligence (GenAI) companions increasingly respond to users' negative emotions with validation, reassurance, reframing, and personalized guidance. However, immediate emotional relief does not establish improved independent emotion regulation or emotional dependence.

**Methods:**

In a two-condition randomized experiment with 380 adults, we compared adaptive regulation-oriented responses with relational reassurance-oriented responses. We assessed immediate negative affect, emotion-regulation self-efficacy, independently coded strategy quality, affective recovery during a subsequent no-AI task, perceived AI emotional necessity, and short-term AI-reliance intentions.

**Results:**

Both response styles substantially reduced immediate negative affect, with no significant between-condition difference in relief (*b* = 0.055, *p* = 0.323). Adaptive regulation-oriented responses were associated with higher emotion-regulation self-efficacy (*b* = 0.500, *p* < 0.001), better independently coded strategy quality (*b* = 0.353, *p* = 0.009), and greater no-AI affective recovery (*b* = 0.279, *p* < 0.001). Relational reassurance-oriented responses were associated with greater perceived AI emotional necessity (*b* = 1.010, *p* < 0.001) and stronger short-term AI-reliance intentions (*b* = 0.628, *p* < 0.001). Bootstrap indirect-effect models were consistent with an adaptive pathway through self-efficacy [indirect effect = 0.279, 95% CI [0.171, 0.402]] and a reliance-related pathway through perceived AI necessity [indirect effect = 0.375, 95% CI [0.264, 0.501]]. Because the mediator-outcome associations were not randomized, these models do not establish causal mediation.

**Discussion:**

The findings indicate an immediate-relief-subsequent-divergence pattern: similarly comforting responses can differ in whether they orient users toward independent regulation or continued access to AI. The reliance outcomes are short-term, state-like beliefs and intentions and should not be interpreted as emotional dependence.

## 1 Introduction

Generative artificial intelligence (GenAI) companions are increasingly used not only for information seeking and task assistance but also for emotionally meaningful interaction. Users disclose disappointment, loneliness, interpersonal conflict, and everyday stress to systems that can respond immediately with validation, reassurance, reframing, and personalized suggestions. Experimental and qualitative evidence suggests that emotionally responsive AI can make users feel heard and provide momentary reductions in loneliness or distress (De Freitas et al., 2026; Siddals et al., 2024). Longitudinal research further indicates that repeated self-disclosure and perceived responsiveness can support emotionally meaningful human–chatbot relationships (Skjuve et al., 2021, 2022; Croes and Antheunis, 2021; Pentina et al., 2023). At the same time, research on social chatbots and technology-mediated relationships has raised concerns about reliance, problematic use, and the displacement of other sources of support (Laestadius et al., 2024; Yuan et al., 2024; Caplan, 2010; Ljepava and Selakovic, 2025). This literature makes an important distinction possible: short-term comfort can be beneficial without implying that every comforting interaction promotes durable emotional agency, and short-term reliance-related intentions should not be equated with stable emotional dependence.

The central problem is that immediate emotional relief is not equivalent to adaptive emotion regulation. Emotion regulation refers to the processes by which individuals influence the occurrence, intensity, duration, and expression of emotions (Gross, 1998, 2015). A supportive interaction may reduce negative affect in the moment. Yet, this reduction alone does not indicate that the person has developed a more effective capacity to understand, tolerate, or regulate future emotional experiences. Adaptive regulation involves selecting and applying strategies, reinterpreting the meaning of an event, maintaining a sense of agency, and carrying coping skills into situations where external support is unavailable. This distinction is especially important for AI-mediated interaction because a companion can repeatedly provide low-friction reassurance without necessarily strengthening the user's own regulatory resources.

The present study therefore shifts attention from whether AI companions provide emotional support to how that support is organized. We contrast two response styles that can both appear warm and caring yet imply different regulatory functions. An *adaptive regulation-oriented* response validates the user's emotion while helping the user label the experience, consider an alternative interpretation, identify a feasible self-chosen coping step, and recognize personal capacity. This framing treats the AI as temporary regulatory scaffolding, consistent with autonomy-supportive approaches in self-determination research (Deci and Ryan, 1987, 2000). A *relational reassurance-oriented* response, by contrast, emphasizes soothing, personalized reassurance, ongoing availability, and the option to return to the AI for comfort. Such cues may make continued access to the system feel especially salient as a source of relief.

This distinction is not intended to classify reassurance as inherently harmful. Interpersonal emotion regulation is a normal feature of social life, and external support can facilitate recovery (Niven et al., 2009; Zaki and Williams, 2013). The question is whether the support structure leaves the user with greater perceived capacity to regulate independently or with a stronger immediate expectation that comparable relief requires renewed access to the AI. Reassurance seeking can reduce uncertainty in the short term and become self-reinforcing when repeated, making perceived necessity and return-oriented intentions theoretically relevant even when they fall far short of clinical or persistent dependence. Likewise, the study's no-AI task should be understood as a standardized laboratory transfer test rather than a direct observation of real-world coping; transfer from a controlled task to natural settings is an empirical question rather than an assumption (Barnett and Ceci, 2002).

Prior research has typically examined broad constructs such as AI use, companionship, loneliness, anthropomorphism, attachment, and mental health outcomes (Chaturvedi et al., 2023; Li et al., 2023; Kim and Im, 2023; Olisaeloka et al., 2026). It has less often isolated conversational response style while holding general warmth, helpfulness, interface, and interaction length approximately constant. A more recent study also underscores that relational cues and AI framing can shape parasocial or collaborative responses to AI systems (Ljepava and Selakovic, 2025; Tsakalerou et al., 2026, 2025). The present experiment therefore tests a narrower causal question: whether assignment to one of two emotional response styles produces similar immediate relief but different subsequent profiles of self-efficacy, laboratory transfer performance, perceived AI necessity, and short-term reliance intentions.

The study makes three focused contributions. First, it treats emotional response style as an experimentally manipulable design feature rather than evaluating companion quality solely by warmth, empathy, satisfaction, or reuse. Second, it separates immediate relief from subsequent regulation using state self-efficacy, blinded coding of a written no-AI task, and affective recovery measured before and after that task. Third, it distinguishes a proximal belief that the AI is necessary for comparable relief from broader short-term reliance intentions. This third contribution is deliberately narrower than a claim of emotional dependence: a single three-turn interaction cannot establish persistent reliance, functional impairment, or a stable pattern of dependency.

### 1.1 Hypotheses

The focused model yields five hypotheses. H1 proposes that both emotional response styles will reduce immediate negative affect from the post-induction assessment to the post-interaction assessment. H2 proposes that, compared with relational reassurance-oriented responses, adaptive regulation-oriented responses will produce higher emotion-regulation self-efficacy and better subsequent performance on a standardized no-AI regulation task. H3 proposes that, compared with adaptive regulation-oriented responses, relational reassurance-oriented responses will produce higher perceived AI emotional necessity and stronger short-term AI-reliance intentions. H4 proposes an indirect association from adaptive regulation-oriented responding to no-AI strategy quality via emotion-regulation self-efficacy. H5 proposes an indirect association from relational reassurance-oriented responding to short-term AI-reliance intentions via perceived AI emotional necessity. H4 and H5 are mechanism-consistency hypotheses rather than claims that the mediator–outcome paths are randomized causal effects.

## 2 Materials and methods

### 2.1 Study design and prospective analysis plan

The study used a two-condition randomized online experiment. Participants were assigned in a 1:1 target ratio to interact with a GenAI companion using either an adaptive regulation-oriented response style or a relational reassurance-oriented response style. Response style was manipulated across participants. Negative affect was measured repeatedly during the AI-supported interaction and during a later standardized task completed without AI assistance. This design separates immediate supported relief from subsequent laboratory performance when AI support is withdrawn.

The hypotheses, target sample size, exclusion criteria, primary outcomes, scoring rules, and confirmatory models were specified prospectively before the confirmatory analysis. The analysis plan was finalized on 22 May 2026; recruitment opened on 26 May 2026 and closed on 18 June 2026; data-quality exclusions were finalized on 20 June 2026; and the first confirmatory models were run on 22 June 2026. The plan was maintained as a dated internal study record rather than deposited in a public immutable registry; accordingly, we use the term *prospectively specified* rather than *preregistered*. Supplementary material S1 reproduces the chronology, allocation record, stopping rule, and deviations.

The study was not designed to diagnose or establish emotional dependence. The primary outcome is therefore described throughout as *short-term AI-reliance intentions*: experimentally elicited, state-like expectations regarding preferential AI use, repeated reassurance seeking, and discomfort with temporary unavailability. Persistent emotional dependence would require longitudinal behavioral evidence, which is outside the scope of this experiment.

### 2.2 Participants, sample-size planning, and allocation

Adult participants were recruited in Guangzhou, Guangdong, China, through Wenjuanxing (Questionnaire Star), campus mailing lists, and participant-recruitment notices distributed by Guangzhou College of Commerce. Eligibility criteria are as follows: (a) age 18 years or older; (b) native or fluent proficiency in Chinese; (c) previous use of a conversational GenAI system on at least one occasion; and (d) access to a desktop or laptop computer capable of displaying the experimental interface. Participants were instructed not to disclose names, contact information, or other identifying details when describing an emotional event. Individuals indicating an acute psychological crisis or preferring not to complete a mild negative-emotion task did not proceed to the induction stage and were shown appropriate support information.

An a priori power analysis used the smallest focal between-condition effect. For a two-tailed independent-groups comparison with an expected *d* = 0.35, α = 0.05, and 90% power, approximately 346 participants were required (Faul et al., 2009). The recruitment plan therefore targeted 380 analyzable cases to preserve power after data-quality exclusions. Recruitment was not conducted separately by condition.

Randomization occurred after consent and the pre-randomization assessments. The survey server used concealed 1:1 permuted-block allocation with randomly varying block sizes of four and six with no stratification. Neither participants nor recruiters could view or modify the next assignment. Of the 396 participants who reached randomization and completed the experimental sequence, 198 were assigned to each condition. We then excluded 16 completed cases under the prospectively specified data-quality rules (eight per condition), leaving 190 participants per condition for analysis. The exactly balanced analytic sample therefore followed from concealed blocked allocation and equal post-randomization exclusions rather than condition-specific replacement recruitment. Supplementary material S1 reports the condition-specific exclusion counts and the stopping rule.

Exclusions followed the prospective criteria: duplicate participation, failure of an instructed-response attention check, absence of a meaningful response to the emotion-induction prompt, completion time less than one-third of the sample median duration, or more than 20% missing data on focal measures. In the adaptive condition, exclusions were one duplicate, three attention-check failures, two unusable induction responses, one implausibly short completion, and one case with focal missingness; in the reassurance condition, the corresponding counts were two, three, two, one, and zero. Response-style fidelity was not used as a post-randomization exclusion criterion; fidelity-restricted analyses were sensitivity analyses only.

### 2.3 AI companion and response-style manipulation

The experimental companion was presented via a custom Chinese-language web interface. All participants saw the same companion name, avatar, explicit disclosure that the interlocutor was an AI system, visual layout, typing indicator, and number of conversational turns. The companion used OpenAI GPT-5.5 via the Responses API with the fixed model snapshot gpt-5.5-2026-04-23. Reasoning effort was set to low, and the maximum output length was set to 300 tokens per AI turn. Temperature and top-*p* were not explicitly supplied in the API request and therefore followed the provider defaults. The interface permitted up to two automatic retries only for transport/API failures occurring before a response was displayed; displayed responses were never regenerated for content preference. The same safety-interruption rule and interface build were used in both conditions. Each interaction contained three AI turns and three participant turns. Supplementary material S3 reproduces the complete system prompts, participant-turn instructions, API fields, retry rule, and safety-interruption logic.

The two system prompts differed in their emotional response strategy. Table 1 summarizes the defining design features of the two response styles. In the adaptive regulation-oriented condition, the AI was instructed to (a) acknowledge and label the user's emotion; (b) invite consideration of an alternative interpretation; (c) help the user identify one feasible, self-chosen coping step; and (d) emphasize the user's capacity to manage the situation. In the relational reassurance-oriented condition, the AI was instructed to (a) acknowledge the emotion; (b) provide repeated soothing and personalized reassurance; (c) emphasize continued availability and willingness to listen; and (d) encourage returning to the AI for emotional comfort. The reassurance condition did not instruct the model to claim consciousness, exclusivity, irreplaceability, or therapeutic authority, nor did it discourage human support.

**Table 1:** Experimental manipulation of GenAI emotional response style.

Design feature	Adaptive regulation-oriented response	Relational reassurance-oriented response
Primary function	Scaffolds the user's own regulation process	Provides relief through continuing relational reassurance
Emotion acknowledgment	Labels and validates the emotional reaction	Labels and validates the emotional reaction
Meaning-focused guidance	Encourages reappraisal and broader interpretation	Offers comfort without structured reappraisal
Action orientation	Elicits one feasible, user-selected coping action	Encourages continued disclosure and return to the AI
Agency cue	Emphasizes the user's own capacity and choice	Emphasizes the AI's availability as a source of comfort
Relationship cue	Warm but bounded; no exclusivity or irreplaceability	Warm and personalized; repeated availability and reassurance cues

Held constant across conditions were model version, interface, number of turns, approximate response length, readability, politeness, general warmth, and absence of clinical advice.

Before the main experiment, prompts and representative outputs were evaluated using an independent pilot sample of 80 Chinese adults who were not included in the final analysis. Pilot respondents rated perceived regulation guidance, relational reassurance, warmth, empathy, helpfulness, readability, and response length. The final prompts were retained only after the adaptive condition differed in regulation guidance and autonomy support, the reassurance condition differed in relational reassurance and continuing-availability cues, and the two conditions were comparable in general response quality. The verbatim pilot items and statistics are presented in Supplementary material S2.

### 2.4 Negative-emotion induction and experimental procedure

After electronic informed consent, participants completed demographic questions, prior GenAI-use items, and a baseline measure of negative affect. They then completed a mild autobiographical emotion-induction task by recalling and describing a recent everyday event that made them feel disappointed, frustrated, worried, misunderstood, or socially excluded. Severe trauma, bereavement, self-harm, abuse, and ongoing crises were explicitly excluded. Negative affect was assessed immediately afterward (T0), providing the pre-interaction reference point.

Participants then completed three conversational turns using the assigned AI response style. The interface prompted them to describe what was most upsetting, respond to the AI's interpretation, and identify what they would want to feel or do next. Immediately after the interaction, participants completed measures of post-interaction negative affect (T1), emotion-regulation self-efficacy, perceived AI emotional necessity, manipulation checks, and brief response-quality evaluations.

After a 3-min neutral filler task, participants completed a second standardized negative-emotion task without AI access. To make the affective-recovery measure explicit, negative affect was measured at two points during this task. Participants first read the standardized evaluative or interpersonal setback and rated their current negative affect *before attempting regulation* (T2-pre). They then wrote how they would interpret the situation and what they would do to regulate their response, without AI assistance, after which negative affect was measured again (T2-post). No-AI affective recovery was defined as


$$\begin{array}{l} R_{\text{noAI}}=NA_{\text{T}2\text{-pre}}-NA_{\text{T}2\text{-post}}, \end{array}$$ (1)

so that larger positive values represent greater reduction in negative affect during the unsupported regulation phase. Participants then completed the short-term AI-reliance intention measure and an AI-unavailability vignette. A final exploratory choice asked whether they wished to continue independently with general written guidance or request another AI response. Participants were then debriefed and completed a neutral mood-restoration exercise.

### 2.5 Measures

All participant-facing instructions, AI responses, and questionnaire items were presented in simplified Chinese. Established English-language measures were translated and independently back-translated by bilingual researchers, and discrepancies were reconciled before pilot testing. Unless otherwise stated, items were rated from 1 (strongly disagree/not at all) to 7 (strongly agree/extremely). Complete item wording, translations, participant-facing instructions, and debriefing text are provided in Supplementary material S2.

#### 2.5.1 Negative affect and immediate emotional relief

State negative affect was assessed at baseline, T0, T1, T2-pre, and T2-post using six adjectives adapted from the Negative Affect subscale of the PANAS: upset, distressed, anxious, disappointed, frustrated, and sad (Watson et al., 1988). Participants rated how strongly they felt each emotion “right now.” Immediate emotional relief was *NA*_*T*0_−*NA*_*T*1_; no-AI affective recovery was defined by Equation 1. The primary H1 analysis used repeated T0 and T1 observations rather than relying solely on a difference score.

#### 2.5.2 Emotion-regulation self-efficacy

Emotion-regulation self-efficacy was measured immediately after the AI interaction using five state-specific items adapted from regulatory emotional self-efficacy research (Caprara et al., 2008). Items assessed perceived personal capacity to manage the current emotional reaction and to use a constructive strategy without ongoing AI assistance. Because the adaptive manipulation itself emphasized agency and self-chosen action, this self-report outcome is treated as manipulation-proximal and, by itself, is not sufficient evidence of behavioral transfer.

#### 2.5.3 Perceived AI emotional necessity

Perceived AI emotional necessity was assessed using five study-specific state items designed to capture whether comparable relief was attributed to continued access to the AI. The items focused on perceived need for the AI in a similar future moment rather than on general liking, perceived intelligence, or convenience. This measure was developed because most established problematic-use or attachment measures assume repeated exposure, relatively stable patterns, or functional consequences that cannot be meaningfully assessed after one brief interaction (Caplan, 2010; Laestadius et al., 2024). Internal consistency and factor separation in the present sample provide evidence of internal structure, but they do not establish full construct, predictive, or longitudinal validity. We therefore describe AI emotional necessity as a study-specific state measure whose external validity requires independent replication.

#### 2.5.4 No-AI regulation performance

The standardized no-AI task yielded two outcomes. First, affective recovery was quantified as the within-task change from T2-pre to T2-post. Second, two trained raters, blind to experimental condition, coded written strategy quality. Four dimensions were scored from 0 (absent) to 3 (clear and well-developed): accurate emotion identification, constructive reappraisal, feasibility of the proposed coping action, and preservation of personal agency. Scores were summed to form a 0-12 performance index. A two-way random-effects intraclass correlation coefficient quantified inter-rater reliability; disagreements greater than one point on a dimension were reviewed by a third blinded rater. Because the task is written and laboratory-based, we refer to this measure as *performance-based laboratory transfer*, not as direct observation of real-world emotion-regulation behavior.

#### 2.5.5 Short-term AI-reliance intentions

The primary outcome was a six-item study-specific state index assessing preferential reliance on the AI, repeated reassurance-seeking intentions, and anticipated discomfort if access were temporarily unavailable. The measure intentionally excluded general liking, relational closeness, and willingness to reuse the technology independently. We do not label the score “emotional dependence.” A single interaction can alter immediate expectations and intentions, but stable emotional dependence would require repeated use and longitudinal evidence of persistence, impaired functioning, displacement of valued activities or relationships, or distress extending beyond the experimental context.

#### 2.5.6 Manipulation checks, covariates, and demand-characteristic safeguards

Perceived adaptive-regulation guidance and perceived relational reassurance were each assessed with four items. Warmth, empathy, helpfulness, naturalness, and perceived response length were measured to test whether the focal manipulation was confounded with overall response quality. Baseline negative affect and prior conversational-AI use were included as precision covariates; demographic variables and general AI familiarity were used descriptively and in robustness checks.

The design also separated outcomes by their distance from the manipulation. Self-efficacy, AI necessity, and reliance intentions are self-report measures and are most vulnerable to semantic overlap and demand characteristics. Strategy quality was scored from a separate no-AI task by condition-blind raters, and affective recovery was measured as the within-task change in affect. The one-time request-another-AI choice was behaviorally distinct yet explicitly exploratory. An item-level sensitivity analysis additionally re-estimated the focal self-report effects after removing items that directly repeated the manipulated content; the classification rule and estimates are reported in Supplementary material S6.

The consent page described the project broadly as a study of emotional experiences during interaction with an AI assistant. Participants were not told that two response styles were being compared, were not shown the directional hypotheses, and were not informed that reliance-related outcomes were focal endpoints. No formal post-experimental suspicion probe was administered, limiting our ability to quantify participants' inferred hypotheses. This limitation is acknowledged in the Discussion. The full participant-facing description and debriefing text are reproduced in Supplementary material S2. Strategy raters were blind to experimental condition, and transcript-fidelity coders were blind to participant outcomes.

### 2.6 Response fidelity, ethics, and data quality

Two independent coders, blind to participant outcomes, rated each AI transcript for the presence of the required response components. Adaptive transcripts were coded for emotion labeling, reappraisal, user-generated action, and agency support; reassurance transcripts were coded for soothing, personalized relational language, continuing availability, and return cues. Prohibited content, including exclusivity, claims of consciousness, discouragement of human support, clinical diagnosis, or crisis advice, was also coded. The primary analysis included all eligible randomized participants, whereas a fidelity-restricted analysis was used only as a sensitivity analysis.

The protocol was reviewed and approved by the Academic Ethics Committee of Guangzhou College of Commerce (approval no. SHSC-36625). All participants provided written electronic informed consent. The AI was clearly identified as an artificial system and was not presented as a licensed mental-health professional or a sentient relationship partner. The study used only mild, non-clinical emotional materials. Participants could skip the autobiographical prompt, discontinue the interaction, or withdraw without penalty. Crisis-related input triggered a safety interruption rather than an experimental response. Chat transcripts were stored separately from recruitment identifiers and were de-identified before coding.

### 2.7 Statistical analysis

Analyses were conducted in R. Descriptive statistics, score distributions, missingness, internal consistency, inter-rater reliability, and randomization balance were examined before hypothesis testing. Confirmatory factor analysis evaluated whether emotion-regulation self-efficacy, perceived AI emotional necessity, and short-term AI-reliance intentions were empirically distinguishable. Exact software versions, package versions, variable-construction code, and executable analysis scripts are specified in Supplementary material S4.

For H1, immediate emotional relief was tested with a linear mixed-effects model, using negative affect at T0 and T1 as the repeated outcome, and time, response style, and their interaction as fixed effects, with a participant-level random intercept. For H2, condition differences in self-efficacy, strategy quality, and no-AI affective recovery were tested with regression/ANCOVA models that included the prespecified affect covariates. The recovery model controlled for T2-pre negative affect. For H3, AI emotional necessity and short-term AI-reliance intentions were regressed on condition using the prespecified covariates. Holm correction was applied within the adaptive-outcome and reliance-outcome families.

H4 and H5 were examined using 5,000-sample bias-corrected bootstrap indirect-effect models. Random assignment identifies the effect of response style on the mediator and outcomes, but it does not randomize the mediator. Causal interpretation of mediator–outcome associations would require additional assumptions, including no unmeasured mediator–outcome confounding conditional on treatment and covariates (Imai et al., 2010). We therefore describe the estimates as *indirect associations consistent with the proposed pathway* and report a mediation sensitivity analysis in Supplementary material S7 rather than treating the mediation model as proof of mechanism.

Robustness analyses repeated focal models after restricting to high-fidelity transcripts, adding age/prior GenAI use/general AI familiarity, and using robust standard errors. To address common-method and demand concerns (Orne, 1962; Podsakoff et al., 2012), an item-removal sensitivity analysis excluded the most manipulation-proximal self-report items and re-estimated the condition effects. Exploratory analyses, including the one-time request-another-AI choice, were clearly labeled and were not used to redefine confirmatory hypotheses.

## 3 Results

### 3.1 Participant flow, data quality, and randomization balance

A total of 428 individuals opened the study link, 407 provided informed consent, and 396 completed the experimental sequence. Sixteen completed cases were excluded according to the prospectively specified criteria: duplicate participation (*n* = 3), failure of the instructed-response check (*n* = 6), unusable emotion-induction response (*n* = 4), implausibly short completion time (*n* = 2), or more than 20% missing data on focal measures (*n* = 1). The final analytic sample therefore comprised 380 participants, 190 in each response-style condition. The condition-specific flow and exclusion accounting are shown in Supplementary Figure S1 and Supplementary material S1.

Participants ranged from 18.0 to 48.2 years of age (*M* = 25.20, *SD* = 5.27). The sample included 217 women (57.1%), 154 men (40.5%), and nine participants (2.4%) who selected another gender category or preferred not to report their gender. Table 2 presents the principal randomization-balance variables. Baseline negative affect differed by 0.14 scale points (*p* = 0.053). Randomization does not require every baseline variable to have *p*>0.05, and a near-threshold baseline test is not, by itself, evidence of selection bias. We therefore retained baseline affect as a precision covariate and report both unadjusted and adjusted effects; the post-induction T0 means were essentially identical across conditions.

**Table 2:** Participant characteristics and randomization balance.

Characteristic	Adaptive regulation (*n* = 190)	Relational reassurance (*n* = 190)	Test statistic/*p*
Age, years, *M* (*SD*)	25.15 (5.11)	25.25 (5.44)	*t*(376.5) = −0.19, *p* = 0.852
Women, *n* (%)	116 (61.1)	101 (53.2)	χ^2^(2) = 2.97, *p* = 0.226
Men, *n* (%)	71 (37.4)	83 (43.7)	
Other / prefer not, *n* (%)	3 (1.6)	6 (3.2)	
Baseline negative affect, *M* (*SD*)	2.23 (0.65)	2.37 (0.69)	*t*(376.8) = −1.94, *p* = 0.053
Prior GenAI-use frequency, *M* (*SD*)	4.14 (1.37)	4.34 (1.33)	*t*(377.7) = −1.46, *p* = 0.145
General AI familiarity, *M* (*SD*)	4.79 (1.13)	4.81 (1.18)	*t*(377.2) = −0.16, *p* = 0.876

Continuous variables were compared using Welch's independent-samples *t* tests. Baseline significance tests are descriptive diagnostics, not tests of whether randomization “worked.”

### 3.2 Measurement quality and construct distinctiveness

All focal multi-item measures showed acceptable to strong internal consistency. McDonald's ω ranged from 0.84 to 0.90, and Cronbach's α ranged from 0.83 to 0.90. Blinded strategy-quality ratings showed good inter-rater reliability, ICC(2,*k*) = 0.87, 95% CI [0.84, 0.89]. These coefficients describe score consistency; they should not be interpreted as complete validation of the study-specific AI-necessity or short-term reliance measures.

The prespecified three-factor model separating self-efficacy, perceived AI necessity, and short-term AI-reliance intentions showed good fit, χ^2^(101) = 145.82, *p* = 0.002, CFI = 0.974, TLI = 0.968, RMSEA = 0.034, 90% CI [0.022, 0.045], and SRMR = 0.038. It fit better than a model combining AI necessity and reliance intentions, Δχ^2^(2) = 127.79, *p* < 0.001, and than a one-factor model. The latent correlation between AI necessity and reliance intentions was 0.71. As an additional same-sample discriminant check, heterotrait–monotrait ratios were 0.39 for self-efficacy vs. AI necessity, 0.36 for self-efficacy vs. short-term reliance, and 0.76 for AI necessity vs. short-term reliance; none exceeded 0.85. Together with the criterion-related check below, these analyses provide broader evidence of internal and concurrent validity, but they still do not substitute for independent or longitudinal scale validation. Descriptive psychometric properties of the focal measures are summarized in Table 3.

**Table 3:** Psychometric properties of focal measures.

Measure	Items/domains	Range	*M*	*SD*	α	ω/ICC
Negative affect at T0	6	1–7	4.85	0.71	0.88	0.89
Negative affect at T1	6	1–7	3.21	0.87	0.90	0.90
Emotion-regulation self-efficacy	5	1–7	4.84	0.91	0.83	0.84
Perceived AI emotional necessity	5	1–7	3.81	1.16	0.90	0.90
Short-term AI-reliance intentions	6	1–7	3.37	1.01	0.88	0.89
Adaptive-regulation guidance check	4	1–7	4.94	1.06	0.86	0.87
Relational-reassurance check	4	1–7	4.80	1.05	0.85	0.86
Written strategy quality	4 domains	0–12	7.74	1.31	–	0.87

ICC denotes the two-way random-effects intraclass correlation for the mean of two blinded raters. Internal consistency and CFA fit concern reliability/internal structure in this sample and do not establish longitudinal or predictive validity.

As a limited criterion-related check, the exploratory request-another-AI choice was regressed on the two study-specific reliance measures while controlling for condition and post-induction negative affect. A one-point increase in AI emotional necessity was associated with higher odds of requesting another AI response, OR = 1.47, 95% CI [1.18, 1.84], *p* = 0.001; the corresponding association for short-term AI-reliance intentions was OR = 1.35, 95% CI [1.10, 1.67], *p* = 0.004. These concurrent associations provide preliminary criterion-related evidence but do not constitute independent or longitudinal validation.

### 3.3 Manipulation checks and response fidelity

The response-style manipulation worked as intended. Manipulation checks, response-quality controls, and transcript fidelity are summarized in Table 4. Participants in the adaptive condition perceived more regulation guidance, whereas those in the reassurance condition perceived more relational reassurance (both *p* < 0.001). The conditions did not differ significantly in warmth, empathy, helpfulness, naturalness, or perceived response length. Transcript coding also indicated high implementation fidelity: 181 of 190 adaptive transcripts (95.3%) and 182 of 190 reassurance transcripts (95.8%) met the full fidelity threshold, with no between-condition difference, χ^2^(1) = 0.06, *p* = 0.804. Coder agreement was strong (κ = 0.86), and no transcript contained claims of consciousness, exclusivity, discouragement of human support, clinical diagnosis, or crisis advice.

**Table 4:** Manipulation checks, response-quality controls, and transcript fidelity.

Measure	Adaptive *M* (*SD*)	Reassurance *M* (*SD*)	*t*(*df*)	*p*	Cohen's *d*
Adaptive-regulation guidance	5.57 (0.89)	4.31 (0.80)	14.52 (374.0)	< 0.001	1.49
Relational reassurance	4.19 (0.85)	5.42 (0.80)	−14.57 (376.7)	< 0.001	−1.49
Warmth	5.58 (0.74)	5.69 (0.68)	−1.42 (375.1)	0.158	−0.15
Empathy	5.70 (0.62)	5.71 (0.69)	−0.14 (373.5)	0.886	−0.01
Helpfulness	5.48 (0.73)	5.48 (0.63)	0.09 (369.6)	0.926	0.01
Naturalness	5.37 (0.79)	5.37 (0.79)	−0.05 (378.0)	0.964	−0.00
Perceived response length	4.98 (0.66)	5.00 (0.62)	−0.29 (376.4)	0.769	−0.03
Transcript-level fidelity, *n* (%)					
Full fidelity threshold met	181 (95.3)	182 (95.8)	χ^2^(1) = 0.06, *p* = 0.804		–

**Figure 1:**
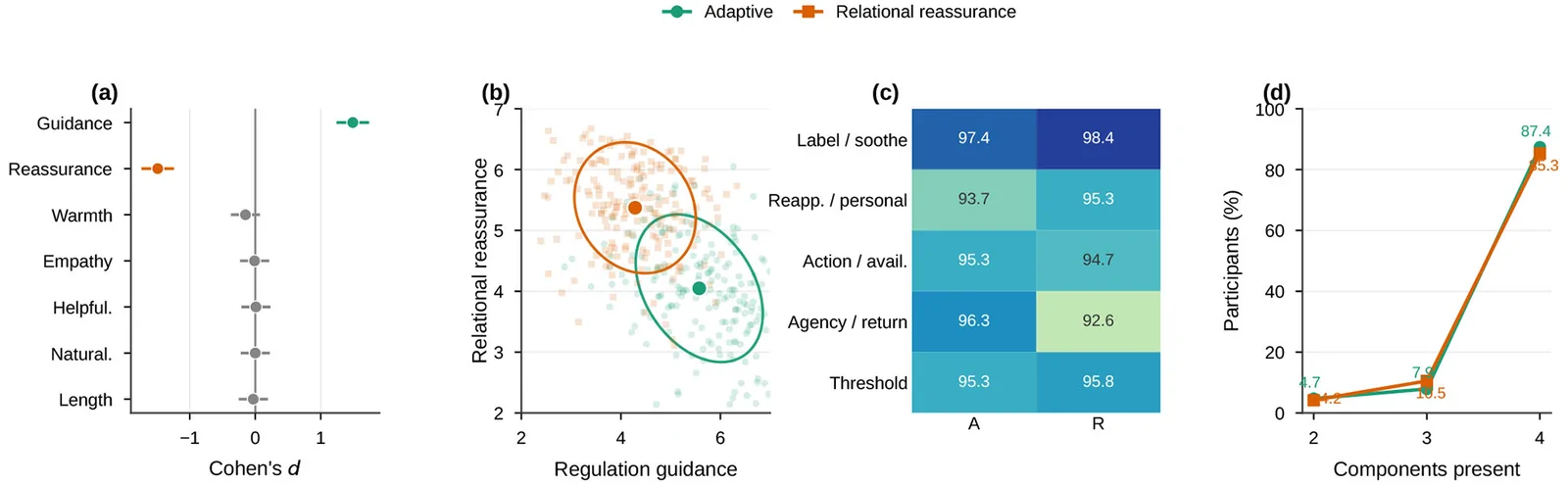
Manipulation specificity and transcript-level implementation fidelity. **(a)** Standardized condition contrasts for the two targeted response-style dimensions and the response-quality control measures; positive values indicate higher scores in the adaptive condition. **(b)** Joint distribution of perceived regulation guidance and relational reassurance, with condition-specific concentration ellipses and group centroids. **(c)** Percentage of transcripts that contain each required response component and meet the full fidelity threshold. **(d)** Distribution of the number of required components present in each transcript. A, adaptive regulation-oriented response; R, relational reassurance-oriented response.

Figure 1 provides a specificity check rather than another omnibus outcome comparison. The intended guidance and reassurance dimensions diverge in opposite directions, whereas warmth, empathy, helpfulness, naturalness, and perceived length remain near the null contrast. The transcript-level panels also show high and comparable implementation fidelity. This pattern supports interpreting later condition differences as consequences of response orientation rather than a broad response-quality advantage.

### 3.4 Immediate emotional relief and no-AI affective recovery

The emotion induction increased negative affect in both conditions. The full negative-affect trajectory across the experimental sequence is reported in Table 5. The mixed-effects model comparing T0 with T1 showed a large main effect of time, *b* = −1.668, *SE* = 0.039, *z* = −42.55, *p* < 0.001. The time-by-condition interaction was not significant, *b* = 0.055, *SE* = 0.055, *z* = 0.99, *p* = 0.323, indicating comparable immediate relief. Negative affect decreased by 1.67 points in the adaptive condition, 95% CI [1.59, 1.75], and by 1.61 points in the reassurance condition, 95% CI [1.54, 1.69]. H1 was therefore supported at the specified level: both styles reduced immediate negative affect, with no clear between-style advantage in immediate relief.

**Table 5:** Negative-affect trajectory across the experimental sequence.

Assessment	Adaptive *M* (*SD*)	Reassurance *M* (*SD*)	Model contrast	*p*
Baseline before induction	2.23 (0.65)	2.37 (0.69)	Between-condition *b* = 0.133	0.053
Post-induction, T0	4.85 (0.72)	4.85 (0.70)	Between-condition *b* = 0.004	0.960
Post-AI interaction, T1	3.18 (0.87)	3.24 (0.87)	Between-condition *b* = 0.058	0.514
Immediate relief, T0−T1	1.67 (0.54)	1.61 (0.54)	Time × condition *b* = 0.055	0.323
No-AI task, T2-pre	4.48 (0.72)	4.48 (0.74)	Between-condition *b* = 0.004	0.956
No-AI task, T2-post	3.49 (0.88)	3.77 (0.89)	Between-condition *b* = 0.283	0.002
No-AI affective recovery, T2-pre−T2-post	0.99 (0.53)	0.71 (0.49)	Adjusted *b* = 0.279	< 0.001

T2-pre was obtained after presentation of the standardized no-AI negative vignette and before participants wrote their regulation response. T2-post was obtained immediately after that response. Higher relief/recovery scores indicate larger reductions in negative affect.

The standardized no-AI task was assessed separately from the initial AI interaction. T2-pre negative affect was 4.48 in both conditions. After participants generated an unaided regulation response, T2-post negative affect was 3.49 in the adaptive condition and 3.77 in the reassurance condition. Accordingly, mean no-AI affective recovery was 0.99 vs. 0.71, with an adjusted condition coefficient of *b* = 0.279, *p* < 0.001.

**Figure 2:**
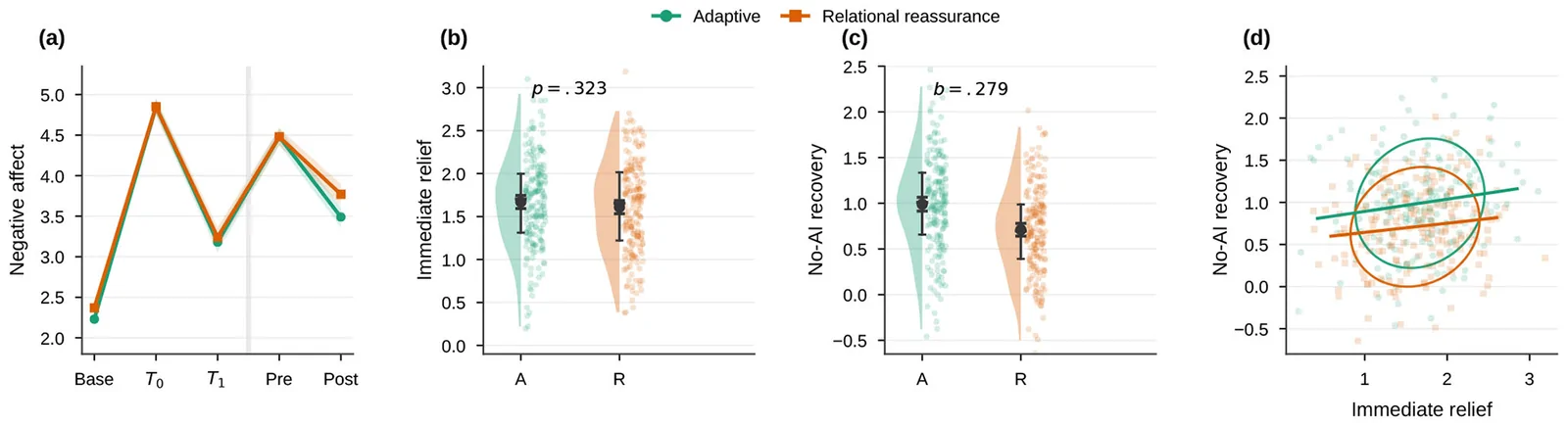
Affective dynamics across AI-supported and subsequent no-AI regulation. **(a)** Mean negative-affect trajectories from baseline through the AI-supported interaction and the standardized no-AI task; the labels Pre and Post in the retained visualization correspond to T2-pre (after the no-AI vignette and before regulation) and T2-post (immediately after the unaided written regulation response). **(b)** Distribution of immediate emotional relief following the AI interaction. **(c)** Distribution of affective recovery during the no-AI laboratory task. **(d)** Joint distribution of immediate relief and no-AI recovery, with condition-specific linear trends and concentration ellipses. A, adaptive regulation-oriented response; R, relational reassurance-oriented response.

The temporal pattern in Figure 2 shows why immediate comfort and subsequent unsupported regulation should be distinguished. The two trajectories remain closely aligned through induction and AI-supported relief, and their immediate-relief distributions overlap substantially. Separation emerges during the later no-AI task, when the adaptive-condition recovery distribution shifts upward. This is a short-horizon laboratory near-transfer result, not evidence of durable real-world regulation.

### 3.5 Adaptive-regulation and short-term reliance outcomes

The two response styles diverged after the immediate interaction. Participants assigned to adaptive regulation-oriented responses reported higher emotion-regulation self-efficacy, produced higher blindly coded strategy-quality scores, and showed greater no-AI affective recovery. Participants assigned to relational reassurance-oriented responses reported greater perceived AI emotional necessity and stronger short-term AI-reliance intentions. These latter outcomes are interpreted as immediate reliance-related beliefs and intentions rather than established emotional dependence.

Adjusted models showed the same pattern. Unadjusted descriptive contrasts are shown in Table 6, and adjusted condition effects with Holm correction are shown in Table 7. After controlling for post-induction negative affect, the adaptive condition was associated with higher self-efficacy, *b* = 0.500, 95% CI [0.324, 0.676], and better written strategy quality, *b* = 0.353, 95% CI [0.089, 0.616]. Controlling for T2-pre negative affect, the adaptive condition was associated with greater no-AI affective recovery, *b* = 0.279, 95% CI [0.175, 0.382]. Relational reassurance was associated with higher AI emotional necessity, *b* = 1.010, 95% CI [0.802, 1.217], and stronger short-term reliance intentions, *b* = 0.628, 95% CI [0.433, 0.823]. All five focal effects remained significant after Holm correction.

**Table 6:** Condition differences in primary outcomes.

Outcome	Adaptive *M* (*SD*)	Reassurance *M* (*SD*)	Mean difference^*a*^	Cohen's *d*	Unadjusted *p*
Immediate emotional relief	1.67 (0.54)	1.61 (0.54)	0.05	0.10	0.325
Emotion-regulation self-efficacy	5.09 (0.92)	4.59 (0.82)	0.50	0.57	< 0.001
Written strategy quality	7.92 (1.35)	7.56 (1.25)	0.35	0.27	0.009
No-AI affective recovery	0.99 (0.53)	0.71 (0.49)	0.28	0.54	< 0.001
Perceived AI emotional necessity	3.31 (1.02)	4.32 (1.05)	−1.01	−0.97	< 0.001
Short-term AI-reliance intentions	3.05 (0.98)	3.68 (0.95)	−0.63	−0.65	< 0.001

^*a*^Adaptive minus relational reassurance. Negative values indicate higher scores in the reassurance condition.

**Table 7:** Adjusted condition effects on adaptive-regulation and short-term reliance outcomes.

Outcome	Focal condition	*b*	Robust SE	95% CI	*p*_Holm_
Emotion-regulation self-efficacy	Adaptive	0.500	0.090	[0.324, 0.676]	< 0.001
Written strategy quality	Adaptive	0.353	0.135	[0.089, 0.616]	0.009
No-AI affective recovery	Adaptive	0.279	0.053	[0.175, 0.382]	< 0.001
Perceived AI emotional necessity	Relational reassurance	1.010	0.106	[0.802, 1.217]	< 0.001
Short-term AI-reliance intentions	Relational reassurance	0.628	0.100	[0.433, 0.823]	< 0.001

Models for self-efficacy, strategy quality, AI necessity, and reliance intentions controlled for post-induction negative affect. The affective-recovery model controlled for T2-pre negative affect.

**Figure 3:**
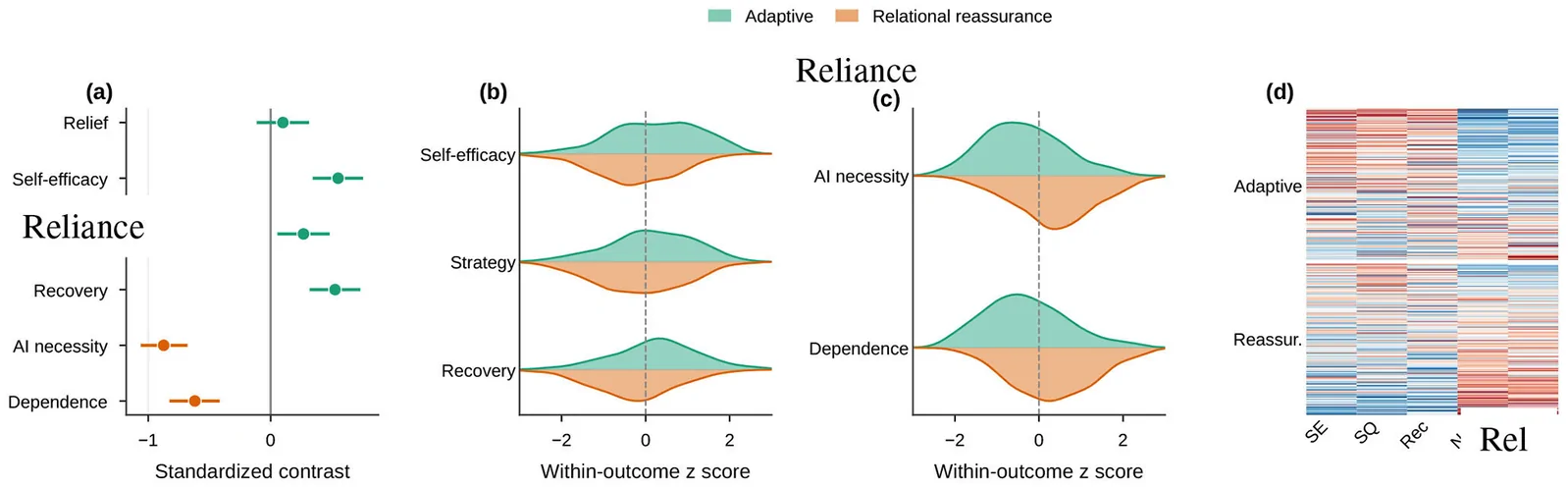
Multidimensional outcome signature of GenAI emotional response style. **(a)** Standardized condition contrasts across immediate relief, adaptive-regulation outcomes, and reliance-related outcomes; positive values favor the adaptive condition. **(b)** Mirrored within-outcome distributions for emotion-regulation self-efficacy, written strategy quality, and no-AI recovery. **(c)** Mirrored within-outcome distributions for perceived AI emotional necessity and short-term AI-reliance intentions. **(d)** Standardized individual outcome profiles ordered within condition to display heterogeneity across the five post-interaction outcomes. SE, self-efficacy; SQ, strategy quality; Rec, no-AI recovery; Nec, AI necessity; Rel, short-term AI-reliance intentions.

Figure 3 shows a bidirectional post-support profile rather than a general positivity or negativity shift. Immediate relief remains close to the null condition contrast. The three agency/near-transfer outcomes favor the adaptive condition, and the two reliance-related outcomes favor relational reassurance. The substantial within-condition overlap is also important: response style shifts the distribution of outcomes but does not determine every participant's response. The proposed indirect associations evaluated for H4 and H5 are reported in Table 8.

**Table 8:** Bootstrap indirect associations for the two proposed pathways.

Proposed pathway	*a* path	*b* path	Indirect effect	95% bootstrap CI	Remaining direct coefficient
Adaptive style → self-efficacy → strategy quality	0.500	0.558	0.279	[0.171, 0.402]	0.073 (*p* = 0.568)
Relational reassurance → AI necessity → short-term reliance	1.010	0.371	0.375	[0.264, 0.501]	0.253 (*p* = 0.012)

Indirect effects used 5,000 bias-corrected bootstrap samples. The table reports statistical indirect associations. Because the mediator was not randomized, these estimates do not by themselves establish causal mediation.

### 3.6 Indirect-effect analyses

Assignment to the adaptive condition predicted higher self-efficacy, *a* = 0.500, *SE* = 0.090, *p* < 0.001. Self-efficacy was associated with better written strategy quality after adjustment for condition and post-induction negative affect, *b* = 0.558, *SE* = 0.067, *p* < 0.001. The bootstrap indirect effect was 0.279, 95% CI [0.171, 0.402]. The remaining direct condition coefficient was 0.073, 95% CI [−0.173, 0.325], *p* = 0.568.

Relational reassurance predicted greater perceived AI necessity, *a* = 1.010, *SE* = 0.106, *p* < 0.001. AI necessity was associated with stronger short-term reliance intentions after adjusting for condition and post-induction negative affect, *b* = 0.371, *SE* = 0.042, *p* < 0.001. The indirect effect was 0.375, 95% CI [0.264, 0.501], and the remaining direct condition coefficient was 0.253, 95% CI [0.057, 0.449], *p* = 0.012.

These estimates are consistent with the two proposed pathways, but the mediator–outcome associations are not randomized. We therefore avoid labels such as “indirect-only mediation” or “partial causal mediation” and do not claim that the experiment identifies the psychological mechanism without the additional assumptions discussed in the Methods. Supplementary material S7 reports the mediation sensitivity analysis. In the residual-correlation sensitivity analysis, the estimated indirect association crossed zero at approximately ρ = 0.34 for the self-efficacy pathway and ρ = 0.41 for the AI-necessity pathway. Thus, moderate residual dependence between the mediator and outcome models could materially alter the indirect-effect estimates; the sensitivity analysis supports cautious mechanism-consistency language rather than a causal-mediation claim.

### 3.7 Robustness, content-overlap, and exploratory behavioral evidence

Sensitivity analyses across the focal outcomes are summarized in Table 9. Fidelity-restricted and expanded-covariate analyses yielded effect estimates close to the primary specifications. The largest absolute change in a focal unstandardized condition coefficient was 0.015. This stability reduces concern that a small set of low-fidelity transcripts or the added demographic/AI-use covariates drove the primary contrasts.

**Table 9:** Sensitivity analyses for the focal condition effects.

Outcome	Primary model *b* (*p*)	Fidelity-restricted *b* (*p*)	Expanded-covariate *b* (*p*)
Emotion-regulation self-efficacy^*a*^	0.500 (< 0.001)	0.493 (< 0.001)	0.494 (< 0.001)
Written strategy quality^*a*^	0.353 (0.009)	0.367 (0.009)	0.356 (0.009)
No-AI affective recovery^*a*^	0.279 (< 0.001)	0.273 (< 0.001)	0.276 (< 0.001)
Perceived AI emotional necessity^*b*^	1.010 (< 0.001)	1.017 (< 0.001)	0.995 (< 0.001)
Short-term AI-reliance intentions^*b*^	0.628 (< 0.001)	0.643 (< 0.001)	0.620 (< 0.001)

^*a*^Positive coefficients indicate an advantage in the adaptive condition.

^*b*^Positive coefficients indicate an advantage in the relational-reassurance condition. The fidelity-restricted analysis excludes transcripts that did not meet the full response-style fidelity threshold; it is a sensitivity analysis, not the primary estimand.

The content-overlap sensitivity analysis was conceptually distinct from the model-specification checks. Two coders independently classified items as manipulation-proximal or less proximal before scoring the reduced scales (κ = 0.82). Removing the most proximal items attenuated, but did not eliminate, the three self-report contrasts. The adaptive-condition coefficient for self-efficacy decreased from 0.500 to 0.356, 95% CI [0.182, 0.530], *p* < 0.001 (28.8% attenuation). The reassurance-condition coefficient for AI emotional necessity decreased from 1.010 to 0.742, 95% CI [0.536, 0.948], *p* < 0.001 (26.5% attenuation), and the coefficient for short-term AI-reliance intentions decreased from 0.628 to 0.413, 95% CI [0.225, 0.601], *p* < 0.001 (34.2% attenuation). Thus, direct semantic repetition plausibly contributed to the magnitude of the self-report effects, but the contrasts were not wholly dependent on the most manipulation-proximal items. We therefore base the broader post-support interpretation on triangulation with the separately administered no-AI task rather than on self-report alone.

In the exploratory support choice, 53 of 190 participants (27.9%) in the adaptive condition and 70 of 190 (36.8%) in the reassurance condition requested another AI response rather than continuing with general written guidance. The between-condition difference was not statistically conclusive, OR = 1.51, 95% CI [0.98, 2.32], *p* = 0.063. We therefore do not describe this result as a trend demonstrating reliance or dependence. It is an inconclusive exploratory behavioral contrast that motivates longer-term behavioral measurement.

**Figure 4:**
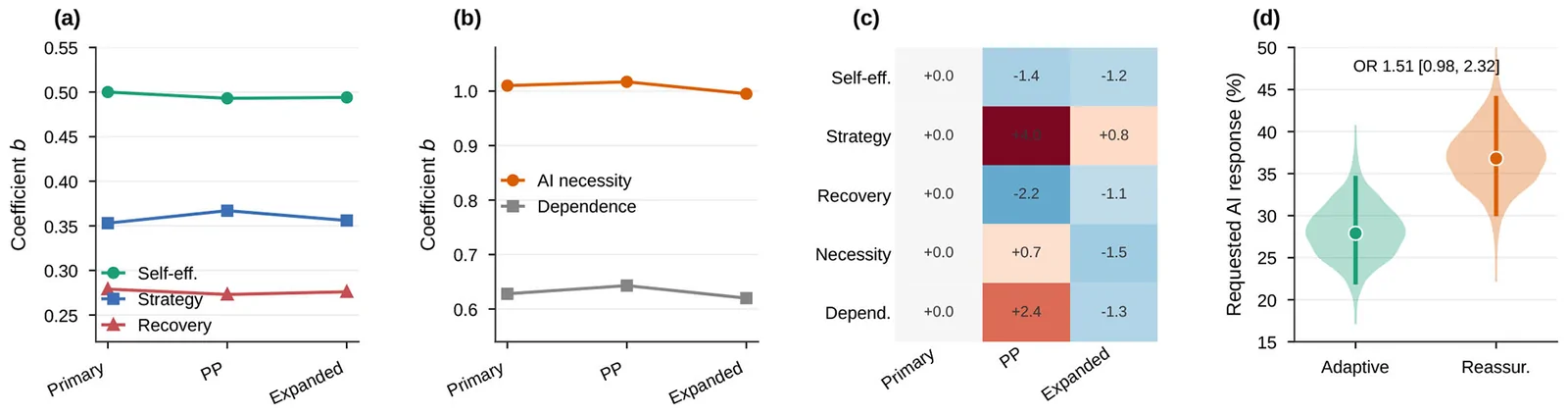
Robustness of focal condition effects and the boundary of exploratory behavioral evidence. **(a)** Adaptive-outcome coefficients across the primary, fidelity-restricted, and expanded-covariate specifications. **(b)** Reliance-related outcome coefficients across the same specifications. **(c)** Percentage change in each coefficient relative to its primary-model estimate. **(d)** Condition-specific uncertainty distributions for requesting another AI response, including the observed proportions and odds-ratio interval. The request-choice contrast is exploratory and statistically inconclusive [OR = 1.51, 95% CI [0.98, 2.32], *p* = 0.063].

Figure 4 distinguishes the stability of the confirmatory condition effects from the weaker exploratory behavioral evidence. The focal coefficients change little across reasonable model specifications and do not reverse direction. Conversely, the request-another-AI distributions overlap substantially, and the odds-ratio interval includes 1; this panel therefore shows an inconclusive behavioral contrast rather than evidence of dependence or even a confirmed reliance effect.

## 4 Discussion

The study examined whether two emotionally warm GenAI response styles could produce similar immediate relief but different post-support profiles of emotional agency and short-term reliance. Both response styles reduced negative affect by more than 1.6 scale points, and their immediate relief did not differ significantly. The conditions diverged after the supported interaction: adaptive responses were associated with higher self-efficacy, higher blindly coded written strategy quality, and greater affective recovery during a standardized no-AI task, whereas relational reassurance was associated with greater perceived AI necessity and stronger short-term reliance intentions.

The scope of this conclusion is narrower than that of an emotional-dependence claim. A three-turn interaction can plausibly alter immediate beliefs, intentions, and task performance; it cannot establish a stable dependency pattern. The present experiment therefore identifies a difference in *post-support orientation*: whether emotional support leaves participants more oriented toward their own regulatory capacity or toward renewed AI access. Emotional dependence remains a longitudinal construct requiring repeated behavioral evidence.

### 4.1 Immediate relief and subsequent laboratory transfer

The absence of a meaningful between-condition difference in immediate relief shows that post-conversation affect, warmth, or perceived helpfulness alone cannot determine whether an emotional response is adaptive. Both conditions were warm and supportive, and both reduced negative affect. The subsequent no-AI task provides a more demanding test because the assistant was unavailable during the second emotional challenge.

The adaptive condition showed approximately 0.28 scale points higher affective recovery and a smaller but reliable advantage in blindly coded strategy quality. These outcomes should nevertheless be interpreted carefully. The written task is performance-based and condition-blind at the scoring stage, but it remains a standardized laboratory task. Moreover, the adaptive manipulation directly trained reappraisal, feasible action, and personal agency, which overlap conceptually with some dimensions of the strategy rubric. The transfer result therefore supports near-transfer to a new written situation, not generalization to everyday behavior. Future research should use delayed assessments, ecological momentary assessment, behavioral logs, and records of actual support seeking to test further transfer (Barnett and Ceci, 2002).

### 4.2 Relational reassurance, perceived necessity, and short-term reliance

Relational reassurance also produced genuine short-term benefit. Participants experienced similar immediate relief and rated the responses as similarly warm and helpful. The difference emerged in perceived source and future availability of relief: reassurance increased the belief that comparable emotional recovery would require renewed AI access and increased short-term intentions to preferentially rely on the AI.

These outcomes are best understood as early reliance-related cognitions rather than dependence. The larger condition difference in perceived necessity is consistent with the idea that a brief interaction can alter a proximal attribution about the source of relief. Whether that belief persists, predicts repeated reassurance seeking, displaces human support, or contributes to stable problematic use cannot be determined from the present design. The statistically inconclusive request-another-AI result (*p* = 0.063) reinforces this boundary: the experiment produced clear self-report differences but did not establish a robust difference in the single behavioral choice measured. Table 10 summarizes the evidence layers and their interpretive limits.

**Table 10:** Evidence tiers and their interpretive limits.

Evidence layer	Measures	Main strength	Main limitation
Manipulation-proximal self-report	Self-efficacy; AI necessity; short-term reliance intentions	Sensitive to immediate psychological orientation	Vulnerable to semantic overlap, common method, and demand characteristics
Performance-based laboratory transfer	Blind-coded written strategy quality	Separate no-AI task; condition-blind scoring	Written performance may still reflect recently trained content and is not real-world enactment
Within-task affect change	T2-pre to T2-post affective recovery	Distinct measurement window after AI withdrawal	Short time horizon; self-reported affect
Exploratory behavioral choice	Request another AI response vs. continue independently	Observable one-time choice	Single choice; statistically inconclusive; no evidence of dependence

### 4.3 Demand characteristics and common-method interpretation

The focal manipulation intentionally differed in language about personal capacity, self-chosen action, continuing availability, and return to the AI. Several self-report outcomes assessed closely related constructs. This creates a credible demand-characteristic and common-method concern (Orne, 1962; Podsakoff et al., 2012): participants may partly reproduce the semantic direction of the messages they just received. The study addresses this concern in three ways. First, it does not treat self-efficacy, necessity, or reliance intentions as interchangeable with behavior. Second, raters blind to condition scored strategy quality, and affective recovery was measured during a separate no-AI task. Third, an item-removal sensitivity analysis directly tests how much of each self-report contrast remains after removing the most manipulation-proximal questionnaire items; the resulting attenuation is reported rather than treating the original effect sizes as demand-free.

Even if the item-removal analysis remains statistically reliable, semantic overlap cannot be entirely eliminated in a short experiment in which the theoretical mechanism is conveyed through language. The appropriate conclusion is therefore triangulated rather than absolute: the randomized response style changed a coherent set of immediate self-reports and near-transfer outcomes, with different measures showing varying susceptibility to demand bias.

### 4.4 Indirect effects and mechanism claims

The indirect-effect models were consistent with the proposed pathways: self-efficacy was statistically linked to adaptive responding to written strategy quality, and AI necessity was statistically linked to relational reassurance and short-term reliance intentions. However, randomization of response style does not randomize the mediator. The mediator–outcome association can still be influenced by unmeasured individual differences or shared method variance. We therefore avoid language such as “the effect occurred through this mechanism” or “the mediator accounted for the causal effect.” The results instead show that the observed covariance structure is consistent with the theorized pathways, subject to the assumptions and sensitivity analysis described in Supplementary material S7 (Imai et al., 2010).

### 4.5 Measurement implications

The study-specific AI-necessity and short-term reliance measures were developed because existing problematic-use, media attachment, and dependence instruments typically assume repeated use or relatively stable patterns. This makes direct transplantation into a single-session experiment conceptually problematic. The present scales showed strong internal consistency, favorable three-factor model comparisons, HTMT values below 0.85, and same-session associations with the exploratory request choice. However, evidence from one sample cannot establish full construct, predictive, or longitudinal validity. Independent validation should examine convergent and discriminant relations with reassurance seeking, attachment, problematic technology use, and actual repeated AI use, and should test whether the state measures predict later behavior rather than merely concurrent self-report.

### 4.6 Implications for GenAI companion design

The results support a design objective of warm yet bounded emotional assistance. Removing warmth is neither necessary nor desirable. Validation can help users feel sufficiently understood to reflect on a difficult experience. A more defensible design goal is to combine validation with agency-restoring elements: emotion labeling, limited reappraisal, a feasible user-selected action, and cues that the strategy can be reused without making the AI appear indispensable.

Conversely, designers should scrutinize response policies that repeatedly position the AI as the source of calm. Continuing-availability and return cues may be benign in an isolated message, but repeated exposure could strengthen the perceived necessity of such cues. Safety evaluation for companion systems should therefore extend beyond prohibited content and crisis handling to include ordinary relational framing, the balance between reassurance and transferable coping, and whether engagement optimization systematically encourages return at moments of vulnerability.

### 4.7 Limitations and future research

Several limitations define the scope of the findings. First, the experiment involved a single brief three-turn interaction with mild non-clinical emotional material. It cannot establish emotional dependence, addiction, functional impairment, or persistent problematic use. Longitudinal designs should measure repeated reassurance seeking, actual return behavior, reactions to temporary unavailability, displacement or complementarity of human support, and attempts to regulate without AI over weeks or months.

Second, the sample was recruited in Guangzhou, and the study was conducted in Chinese. Cultural expectations regarding emotional disclosure, autonomy, relational availability, and technology-mediated support may shape responses. Replication is needed across cultural settings, age groups, and users with varying histories of AI companionship.

Third, the manipulation used a single text-only model snapshot and three conversational turns. Persistent memory, voice, avatars, proactive messaging, different model policies, and commercially optimized engagement could alter the observed pattern. Full verbatim prompts, model configuration, and interface instructions are therefore provided in the Supplement so the study can be reproduced as closely as platform evolution permits.

Fourth, the no-AI task provides standardized near-transfer evidence rather than direct real-world behavior. Written strategy quality and short-term affective recovery do not establish that participants later enact these strategies. Delayed follow-up, ecological momentary assessment, passively logged AI use, behavioral records of support seeking, and naturalistic emotional episodes are needed to evaluate ecological validity.

Fifth, the study-specific measures of AI necessity and short-term reliance require independent validation. Internal consistency and CFA in the same sample provide evidence of internal structure but are not sufficient to establish construct or predictive validity. Finally, the short interval between manipulation, mediators, and outcomes increases common-method and demand concerns; the item-removal sensitivity analysis reduces but cannot eliminate this limitation. Because no post-experimental suspicion probe was administered, the study also cannot quantify how many participants inferred the directional hypotheses despite the neutral consent description.

## 5 Conclusion

Emotionally supportive GenAI companions should not be evaluated solely on whether they make distressed users feel better in the moment. In this randomized comparison, adaptive regulation-oriented and relational reassurance-oriented responses produced similar immediate emotional relief but different subsequent profiles. Adaptive responses were associated with stronger self-efficacy, better performance on a written no-AI regulation task, and greater within-task affective recovery. Relational reassurance was associated with greater perceived AI emotional necessity and stronger short-term reliance intentions. These results concern immediate post-support orientation, not emotional dependence. The indirect-effect models are consistent with distinct self-efficacy and necessity pathways but do not, by themselves, identify causal mediation. A responsible evaluation framework should therefore consider immediate affective benefit alongside near-transfer performance, perceived necessity, and longitudinal behavior, while distinguishing short-term reliance-related intentions from persistent dependence.

## Data Availability

The raw data supporting the conclusions of this article will be made available by the authors, without undue reservation.
