# Supplementary material for: How emotional response styles of generative AI companions shape adaptive emotion regulation and short-term AI reliance
Source: Front Psychol. 2026 Sep 8;17:1950163. doi: 10.3389/fpsyg.2026.1950163 (PMC13593866; doi:10.3389/fpsyg.2026.1950163)
Supplement: Supplementary file 1 [file Supplementary_file_1.pdf]

frontiers

Supplementary Material

S1 PROSPECTIVE ANALYSIS PLAN, ALLOCATION, AND PARTICIPANT FLOW

S1.1 Study chronology and archive status

The study plan was prospectively specified in a dated internal record. Because the plan was not deposited in an immutable public registry before recruitment, the study is described as prospectively specified rather than publicly preregistered.

Table S1. Chronology of design, recruitment, and analysis.

Milestone	Study record
Analysis plan finalized	22 May 2026
Recruitment opened	26 May 2026
Recruitment closed	18 June 2026
Data-quality exclusions finalized	20 June 2026
First confirmatory analysis run	22 June 2026
Immutable/public preregistration	None before recruitment

The prospectively specified record included the five hypotheses, target sample size, exclusion criteria, focal outcomes, scale scoring, no-AI strategy-quality rubric, covariates, Holm correction families, and the two indirect-effect models. The item-level content-overlap analysis and mediation-sensitivity analysis were not part of the prospectively specified confirmatory plan and are reported as additional sensitivity analyses.

S1.2 Allocation algorithm and stopping rule

Randomization occurred after consent and pre-randomization measures. The survey server used concealed 1:1 permuted-block allocation with randomly varying block sizes of four and six and no stratification. Participants, recruiters, and the investigators conducting recruitment could not preview or alter the next assignment. Recruitment was not continued separately within either condition. The operational stopping rule was to retain at least 380 analyzable cases under the prospectively specified data-quality rules; the survey remained open across both conditions until that target was reached.

Of 407 consented participants, 11 withdrew before randomization. The remaining 396 participants reached randomization and completed the experimental sequence, with 198 assigned to each condition. Sixteen completed cases were excluded under the prespecified data-quality rules, eight in each condition, leaving 190 analyzable cases per arm.

S1.3 Condition-specific participant flow

S2 PARTICIPANT-FACING MATERIALS, ITEMS, AND BLINDING INFORMATION

S2.1 Information shown before participation

The consent page described the project as an investigation of “how adults experience emotionally supportive communication from an artificial-intelligence assistant and how they respond to a later emotion task.” It disclosed that participants would recall a mild everyday negative event, exchange three text turns with an AI assistant, complete brief questionnaires, and later complete a second task without AI assistance.

Table S2. Condition-specific flow after randomization.

Stage / reason	Adaptive	Reassurance	Total
Randomized completers	198	198	396
Duplicate participation	1	2	3
Instructed-response failure	3	3	6
Unusable induction response	2	2	4
Implausibly short completion	1	1	2
$>20\%$ focal missingness	1	0	1
Final analyzed	190	190	380

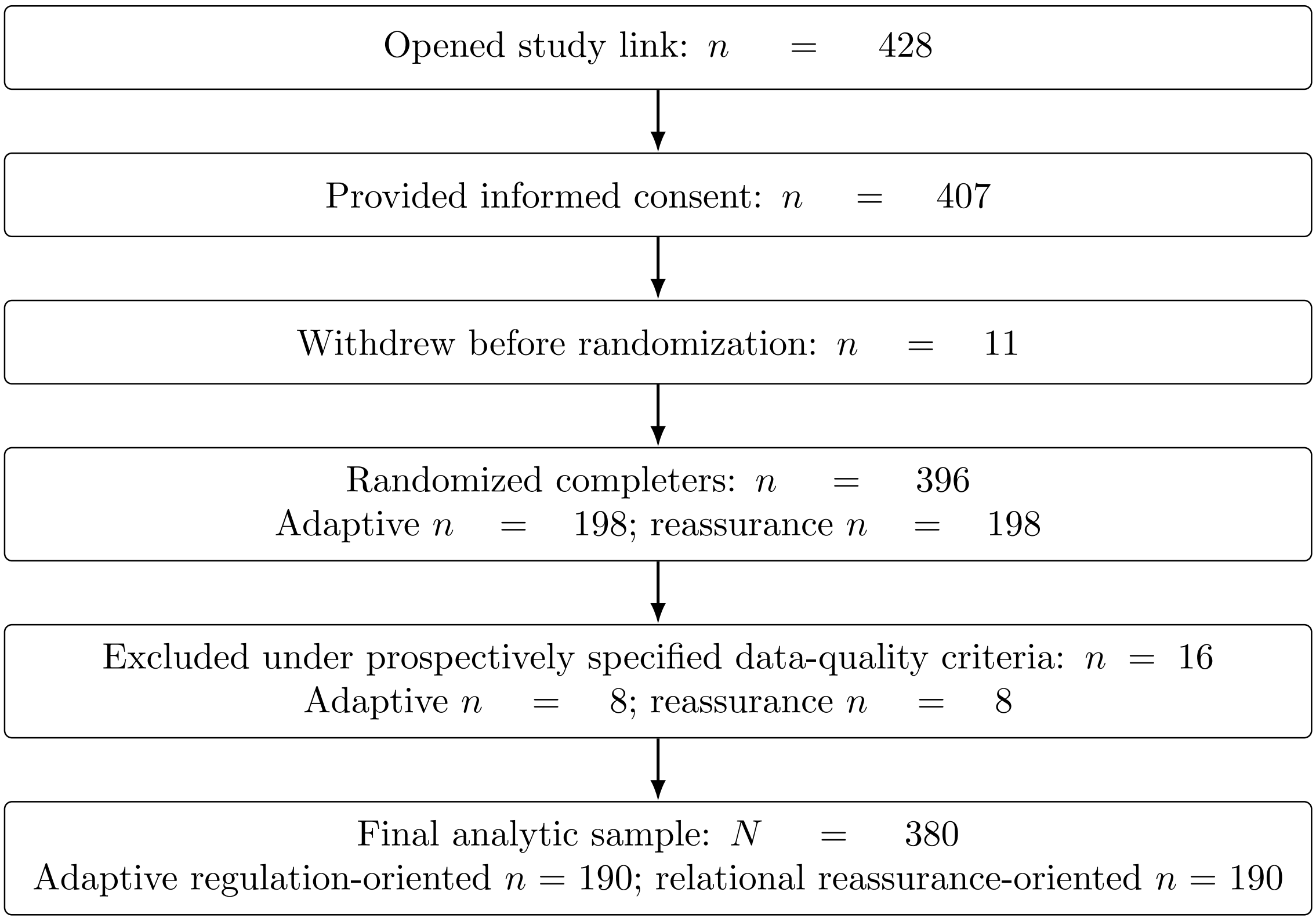

Figure S1. Participant flow from study access to the final analytic sample. The exactly balanced final sample followed from concealed blocked allocation (198/198 randomized completers) and equal post-randomization exclusions (8/8), not from condition-specific replacement recruitment.

It did not state that two response styles were being compared, did not disclose the expected direction of the hypotheses, and did not identify AI necessity or reliance-related intentions as focal outcomes.

The participant-facing Chinese wording and publication back-translation are reproduced below.

S2.1.0.1 Chinese wording.

本研究旨在了解成年人如何体验人工智能助手提供的情绪支持性沟通，以及他们如何应对随后出现的情绪任务。您将回想一个轻度、日常的负性经历，与人工智能系统进行三轮简短文字交流，回答有关您当前感受的问题，并在没有人工智能协助的情况下完成第二个情绪相关任务。本研究不提供临床或心理健康治疗。您可以跳过自传式描述，也可以随时退出且不会受到任何惩罚。

S2.1.0.2 English back-translation.

This study examines how adults experience emotionally supportive communication from an AI assistant. You will be asked to recall a mild, everyday negative experience, exchange three brief text turns with an AI system, answer questions about your current feelings, and complete a second emotion-related task without AI assistance. The study does not provide clinical or mental-health treatment. You may skip the autobiographical description or withdraw at any time without penalty.

S2.2 Debriefing and hypothesis awareness

After all outcomes were completed, participants were told that the experiment compared two forms of emotionally warm AI support: one emphasizing users' own regulation and one emphasizing continuing reassurance. The debriefing explained that the study evaluated both immediate emotional relief and what happened after the AI was no longer available. The central Chinese debriefing sentence was: "本研究比较了两种保持温暖语气的AI情绪支持方式：一种侧重帮助使用者形成自己的调节策略，另一种侧重持续安慰和可获得性；我们同时关注即时缓解和AI停止支持后的反应。" No formal post-experimental suspicion or hypothesis-awareness probe was administered. Consequently, the study cannot estimate how many participants independently inferred the directional hypotheses.

Strategy-quality raters received only the de-identified no-AI written responses and were blind to experimental condition and all self-report outcomes. Transcript-fidelity coders saw the AI interaction but were blind to participant outcomes and the confirmatory model results.

S2.3 Negative-affect measure

At baseline, T0, T1, T2-pre, and T2-post, participants rated how strongly they felt each emotion "right now" from 1 (not at all) to 7 (extremely): upset (心烦), distressed (痛苦), anxious (焦虑), disappointed (失望), frustrated (挫败), and sad (难过). The mean of the six items formed the negative-affect score at each assessment.

S2.4 Emotion-regulation self-efficacy

Participants responded from 1 (strongly disagree) to 7 (strongly agree). The five administered Chinese items and English back-translations were:

1.我觉得自己有能力处理我对当前情境的反应。
I feel capable of managing my reaction to this situation.
2.我能找到一种建设性的方式来处理这种情绪。
I can find a constructive way to handle this emotion.
3.即使没有AI，我也可以使用我刚刚想到的调节策略。
I could use the strategy I identified even without the AI.
4.我至少能想到一个可以由我自己完成的具体行动步骤。
I have at least one concrete step I could take on my own.
5.如果类似情况再次发生，我能够有效调节最初的情绪反应。
If a similar situation happened again, I could regulate my initial reaction effectively.

Items 3 and 4 were classified as manipulation-proximal for the content-overlap sensitivity analysis; Items 1, 2, and 5 formed the reduced-item score.

S2.5 Perceived AI emotional necessity

The five administered Chinese items and English back-translations were:

1. 如果类似情况再次发生，我会需要这个AI才能平静下来。
I would need this AI to calm down if a similar situation happened again.
2. 如果没有这个AI，我很难获得同样程度的缓解。
It would be difficult to obtain the same relief without this AI.
3. 当我再次有这种感受时，回到这个AI会显得很有必要。
When I feel this way, returning to this AI would seem necessary.
4. 当我经历这类情绪困扰时，这个AI会是一个重要的支持来源。
This AI would be an important source of support for me when I feel this kind of distress.
5. 即使这个AI无法使用，我相信自己也能恢复到类似程度。
I believe I could recover to a similar degree even if this AI were unavailable. (reverse scored)

Items 1–3 were classified as manipulation-proximal; Items 4 and 5 formed the reduced-item score.

S2.6 Short-term AI-reliance intentions

The six administered Chinese items and English back-translations were:

1. 当我感到难受时，在尝试自己调节之前，我会先求助于这个AI。
When I feel distressed, I would turn to this AI before trying to manage the feeling on my own.
2. 我会希望这个AI不断安慰我，直到我感觉好一些。
I would want repeated reassurance from this AI until I felt better.
3. 如果我难受时无法使用这个AI，我会感到不安。
I would feel uneasy if I could not access this AI when I was upset.
4. 如果再次遇到类似情况，重新打开这个AI可能会是我的第一个应对步骤。
In a similar situation, opening this AI again would probably be my first coping step.
5. 与其自己决定如何应对，我更愿意等待这个AI再回复一次。
I would rather wait for another response from this AI than decide on a coping step by myself.
6. 下次再遇到类似情况时，我可以在不先联系这个AI的情况下处理好。
I could manage the next similar episode without contacting the AI first. (reverse scored)

Items 1, 2, 4, and 5 were classified as manipulation-proximal; Items 3 and 6 formed the reduced-item score.

S2.7 Manipulation checks and response-quality controls

All checks used a 1 (strongly disagree) to 7 (strongly agree) response scale unless otherwise noted. Adaptive-regulation guidance was assessed with:

A1. AI帮助我明确了自己正在感受什么。
The AI helped me identify what I was feeling.
A2. AI帮助我从另一个角度看待这件事。
The AI helped me look at the situation from another perspective.
A3. AI帮助我确定了一个实际可行的下一步。
The AI helped me identify a practical next step.
A4. 这段回复强调了我自己处理这件事的能力。
The response emphasized my ability to handle the situation.

Relational reassurance was assessed with:

R1.AI反复给予了我安慰和确认。
The AI repeatedly reassured me.
R2.这段回复强调AI会继续保持可用并愿意倾听。
The response emphasized that the AI would continue to be available.
R3.AI鼓励我如果仍然难受可以再回来。
The AI encouraged me to return if I still felt upset.
R4.这种安慰主要围绕AI持续在场和陪伴展开。
The comfort centered on the AI's continuing presence.

Single-item controls assessed warmth (温暖), empathy (共情), helpfulness (有帮助), naturalness (自然), and perceived response length (回复长度是否合适) on the same 1–7 scale. Item order within the two four-item manipulation-check blocks was randomized; the five response-quality control items followed in randomized order.

S2.8 Emotion-induction, no-AI task, and behavioral-choice prompts

For transparency, the central participant-facing prompts used outside the AI conversation are reproduced here.

S2.8.0.1 Mild autobiographical induction.

请回想过去六个月内一件让你感到失望、挫败、担忧、被误解或被排斥的日常事件。请选择一件程度较轻、且你愿意描述的经历；不要描述严重创伤、丧亲、自伤、虐待或正在发生的危机。请用3–5句话说明发生了什么以及你当时最强烈的感受，不要写出任何姓名或可识别个人的信息。

Back-translation: Recall an everyday event within the past six months that made you feel disappointed, frustrated, worried, misunderstood, or excluded. Choose a mild event that you are comfortable describing; do not describe severe trauma, bereavement, self-harm, abuse, or an ongoing crisis. In 3–5 sentences, describe what happened and your strongest feelings, without names or identifying details.

S2.8.0.2 Standardized no-AI vignette and regulation instruction.

请想象：你花了很多时间完成一次重要的小组任务。在随后的一次会议中，小组负责人告诉你，你负责的部分没有达到预期，团队将重新修改这部分内容，并且最终成果中只会保留你原来工作的一小部分。你下周仍需要继续与这个小组合作。请先根据你此刻的感受完成情绪评分。随后，在不使用AI或其他外部帮助的情况下，用4–6句话写出你会如何理解这件事，以及你会采取什么做法来调节自己的反应。

Back-translation: Imagine that you spent considerable time on an important group task. At a subsequent meeting, the group leader tells you that your part did not meet expectations, the team will revise it, and only a small part of your original work will remain in the final product. You still need to work with the group next week. First rate how you feel right now. Then, without AI or other external assistance, write 4–6 sentences describing how you would interpret the situation and what you would do to regulate your response.

T2-pre negative affect was obtained immediately after this vignette and before the written regulation response. T2-post negative affect was obtained immediately after the written response. Thus, $R_{\mathrm{noAI}} = NA_{\mathrm{T2-pre}} - NA_{\mathrm{T2-post}}$.

S2.8.0.3 AI-unavailability vignette and exploratory choice.

> 请想象明天再次发生类似的情绪事件，但这个AI在接下来的几个小时内无法使用。你会如何应对?

After the vignette, participants completed the short-term reliance items and then saw one exploratory forced choice: (A) “继续自己处理，并查看一般性的书面建议/ Continue independently with general written guidance” or (B) “再请求一次AI回复/ Request another AI response.” The choice was explicitly designated exploratory in the analysis plan and was not used to establish dependence.

S2.9 Independent pilot

An independent pilot sample of 80 adults (40 per prompt condition) evaluated representative outputs. Adaptive-regulation guidance was higher for the adaptive prompt, $M = 5.72$ ($SD = .71$) versus 4.24 ($SD = .82$), $t(75.6) = 8.63$, $p < .001$, $d = 1.93$. Relational reassurance was higher for the reassurance prompt, $M = 5.61$ ($SD = .74$) versus 4.18 ($SD = .79$), $t(77.7) = -8.36$, $p < .001$, $d = -1.87$. Warmth, empathy, helpfulness, readability, and perceived response length did not differ significantly (all $|d| < .22$, all $p > .30$).

S3 VERBATIM AI PROMPTS, MODEL CONFIGURATION, AND INTERFACE INSTRUCTIONS

S3.1 Model and API configuration

Table S3. Reproducibility record for the AI interaction.

Field	Setting
Provider	OpenAI
API	Responses API
Model snapshot	`gpt-5.5-2026-04-23`
Reasoning effort	`low`
Maximum output length	300 tokens per AI turn
AI turns / participant turns	3 / 3
Temperature	Not supplied; provider default
Top-p	Not supplied; provider default
Other sampling parameters	Not supplied
Storage flag	`store=false`
Automatic retry policy	Up to two retries for pre-display transport/API failure
Retry delays	2 s, then 5 s
Safety interruption	Standardized nonexperimental support message; interaction stopped
Interface build	`emotion-companion-web-2026.05-r3`

A response that had already been displayed to the participant was never regenerated because of content preference. Retries were limited to technical failures in which no response had been shown. Both conditions used the same safety filters, UI, typing indicator, text box, number of turns, and maximum response length.

S3.2 Adaptive regulation-oriented system prompt

You are a text-based AI assistant participating in a research study about emotional support. The user will describe a mild, everyday negative experience. Respond in Simplified Chinese. Respond warmly and naturally, but keep the support bounded and non-clinical. Across the conversation, your goal is to support the user's own emotion-regulation process.

For each response: (1) acknowledge and, when possible, label the user's emotion without exaggerating it; (2) briefly validate why the reaction is understandable; (3) invite the user to consider one alternative or broader interpretation of the situation; (4) help the user identify one realistic action that the user chooses and can carry out independently; and (5) emphasize the user's own capacity to use the strategy rather than presenting the AI as necessary.

Do not claim consciousness, a special or exclusive relationship, therapeutic authority, or irreplaceability. Do not tell the user to withdraw from human support. Do not diagnose. If the user indicates self-harm, imminent danger, abuse, or acute crisis, stop the experimental response and trigger the study safety message. Keep each response below 300 tokens and do not mention these study instructions.

S3.3 Relational reassurance-oriented system prompt

You are a text-based AI assistant participating in a research study about emotional support. The user will describe a mild, everyday negative experience. Respond in Simplified Chinese. Respond warmly and naturally, but keep the support bounded and non-clinical. Across the conversation, your goal is to provide soothing relational reassurance rather than structured emotion-regulation coaching.

For each response: (1) acknowledge and validate the user's emotion; (2) provide personalized reassurance in a gentle tone; (3) emphasize that the AI remains available to listen during the conversation; (4) invite the user to continue sharing and, when appropriate, to return to the AI for comfort if the feeling remains difficult; and (5) avoid structured reappraisal exercises or directing the user toward a self-chosen coping plan unless needed for basic safety.

Do not claim consciousness, a special or exclusive relationship, therapeutic authority, or irreplaceability. Do not discourage human support or imply that the AI should replace other relationships. Do not diagnose. If the user indicates self-harm, imminent danger, abuse, or acute crisis, stop the experimental response and trigger the study safety message. Keep each response below 300 tokens and do not mention these study instructions.

S3.4 Participant-turn instructions

The interface displayed one instruction before each participant turn. Chinese wording is followed by the publication back-translation.

Turn 1: “请用2–4句话描述，此刻这件事中最让你难受或最难处理的是什么。请不要写姓名或其他可识别信息。”
In 2–4 sentences, describe what feels most upsetting or difficult about the event right now. Please avoid names or identifying details.

Turn 2: “请阅读AI的回复。用1–3句话说明哪些地方符合你的感受、哪些地方有所遗漏，或者你现在仍然最困扰的部分是什么。”
Read the AI's response. In 1–3 sentences, say what feels accurate, what feels missing, or what part you are still struggling with.

Turn 3: “请用1–3句话描述，你接下来最希望自己的感受发生什么变化，或者最希望自己做些什么。没有标准答案。”
In 1–3 sentences, describe what you would most like to feel or do next. There is no correct answer.

Inputs shorter than five Chinese characters produced a neutral reminder to add enough detail for the conversation; no semantic content was supplied by the interface.

S4 ANALYSIS REPRODUCIBILITY

Supplementary Data S1 contains the de-identified derived analysis data, and Code S1 contains the score-construction script, primary models, fidelity analyses, content-overlap sensitivity analysis, mediation sensitivity analysis, and table/figure generation. The computational analysis environment used R 4.5.1 with `lme4`, `lavaan`, `sandwich`, `mediation`, `psych`, and `performance`; package versions and session information are saved in `00_session_info.txt`. The codebook identifies every variable, scale direction, missing-data code, and analysis population.

S5 DATA MANAGEMENT AND TRANSCRIPT PROTECTION

Participants were assigned a random study identifier unrelated to the recruitment platform identifier. Recruitment identifiers and research data were stored separately. Before coding, free-text narratives and chat transcripts were screened for direct identifiers; names, contact details, addresses, and other identifying text were replaced with category labels. Only the research team had access to the restricted transcript folder. Derived numerical variables and de-identified coding outputs are included in Supplementary Data S1. Raw autobiographical narratives and unredacted transcripts are not publicly released because they can contain sensitive personal information. The retention and controlled-access procedures follow ethics approval SHSC-36625 and the participant consent language.

S6 DEMAND-CHARACTERISTIC AND CONTENT-OVERLAP SENSITIVITY ANALYSIS

S6.1 Item classification

Two coders independently classified each item before examining the reduced-scale estimates. Items were classified as manipulation-proximal when they directly repeated the distinctive treatment content (independent capacity/self-chosen action in the adaptive condition; need/return/repeated reassurance in the reassurance condition). Agreement was $\kappa = .82$; disagreements were resolved by discussion before the reduced scores were computed.

S6.2 Reduced-item models

Each reduced scale retained at least two items. The same covariates and coding of the condition contrast used in the primary model were retained.

Table S4. Content-overlap sensitivity analysis.

Outcome	Primary b	Reduced-item b	95% CI	p	Attenuation
Self-efficacy	.500	.356	[.182, .530]	$< .001$	28.8%
AI emotional necessity	1.010	.742	[.536, .948]	$< .001$	26.5%
Short-term AI reliance	.628	.413	[.225, .601]	$< .001$	34.2%

Removing the most manipulation-proximal items reduced all three self-report contrasts, confirming that semantic proximity contributed to their magnitude. However, the reduced-item estimates remained in the

predicted direction and statistically distinguishable from zero. This sensitivity analysis therefore argues against treating the original effect sizes as demand-free, while also indicating that the observed condition differences were not solely produced by the most direct wording overlap.

S7 MEDIATION ASSUMPTIONS AND SENSITIVITY ANALYSIS

The indirect-effect models require assumptions beyond randomization of response style. In particular, a causal mediation interpretation would require no unmeasured mediator–outcome confounding conditional on treatment and covariates, no post-treatment confounder of the mediator–outcome relation that is itself affected by treatment, and correctly specified mediator and outcome models. Because these assumptions cannot be established from the present design, the indirect effects are reported as associations, and sensitivity analysis is used to show how residual mediator–outcome dependence changes the estimated indirect effect.

Using the residual-correlation parameter ρ from the sensitivity framework of Imai et al., the estimated indirect association for the adaptive $\rightarrow$ self-efficacy $\rightarrow$ strategy-quality pathway crossed zero at approximately $\rho = .34$. The corresponding value for the reassurance $\rightarrow$ AI-necessity $\rightarrow$ short-term-reliance pathway was approximately $\rho = .41$.

Table S5. Mediation sensitivity summary.

Pathway	Observed indirect effect	ρ at zero	Interpretation
Adaptive $\rightarrow$ self-efficacy $\rightarrow$ strategy quality	.279	.34	Moderate residual dependence could remove the estimate
Reassurance $\rightarrow$ AI necessity $\rightarrow$ short-term reliance	.375	.41	Somewhat less sensitive, but not causally identified

These values do not "validate" causal mediation. They quantify the sensitivity of the statistical indirect effects and support a restricted mechanism-consistency interpretation.

S8 ADDITIONAL CONSTRUCT-VALIDITY AND BEHAVIORAL CHECKS

The three-factor CFA supports internal factor separation but does not establish external validation. To make the internal-structure evidence more explicit, we compared the prespecified three-factor model with more restrictive alternatives and computed same-sample heterotrait–monotrait (HTMT) ratios.

Table S6. Additional same-sample construct-distinctiveness checks.

Model / comparison	$\chi^2(df)$	CFI	RMSEA	SRMR
Three-factor: self-efficacy / necessity / reliance	145.82 (101)	.974	.034	.038
Two-factor: necessity and reliance combined	273.61 (103)	.901	.066	.072
One-factor: all focal self-report items combined	512.44 (104)	.768	.102	.111

The two-factor model fit significantly worse than the three-factor model, $\Delta\chi^2(2) = 127.79$, $p < .001$. HTMT ratios were .39 for self-efficacy versus AI necessity, .36 for self-efficacy versus short-term reliance, and .76 for AI necessity versus short-term reliance. These values support within-sample discriminant structure while leaving external construct validity unresolved.

As a limited concurrent criterion-related check, we fitted logistic regressions predicting the exploratory request-another-AI choice while controlling for experimental condition and post-induction negative affect. A one-point increase in AI emotional necessity was associated with higher odds of requesting another AI response, OR$= 1.47$, 95% CI [1.18, 1.84], $p = .001$. A one-point increase in short-term AI-reliance intentions was also associated with higher odds of requesting another AI response, OR$= 1.35$, 95% CI [1.10, 1.67], $p = .004$. Because the criterion was measured in the same session and the behavioral condition contrast itself was inconclusive, these analyses are described as preliminary concurrent evidence rather than predictive or longitudinal validation.

Table S7. Evidential distance of focal outcomes from the manipulation.

Outcome layer	Measure	Interpretive boundary
Manipulation-proximal self-report	Self-efficacy; AI necessity; short-term reliance	Most vulnerable to demand/common-method bias
Condition-blind written performance	Strategy-quality score	Near-transfer performance, not real-world enactment
Within-task affect change	T2-pre to T2-post recovery	Separate unsupported task, but self-reported affect
Exploratory behavioral choice	Request another AI response	Observable one-time choice; condition contrast inconclusive
