# Supplementary material for: How emotional response styles of generative AI companions shape adaptive emotion regulation and short-term AI reliance
Source: Front Psychol. 2026 Sep 8;17:1950163. doi: 10.3389/fpsyg.2026.1950163 (PMC13593866; doi:10.3389/fpsyg.2026.1950163)
Supplement: Supplementary file 2 [file Data_Sheet_1.ZIP › AI_Response_Styles_Supplementary_Submission_Clean/Supplementary_Material.pdf]

Supplementary Material

Supplementary Materials S1–S5

Contents

1 Supplementary Material S1: Study Design and Analysis Plan **2**

1.1 Design and objectives . . . 2

1.2 Pilot evaluation and prompt selection . . . 2

1.3 Participant profile and flow . . . 2

1.4 Hypotheses and outcome hierarchy . . . 2

1.5 Eligibility, exclusions, and sample planning . . . 3

1.6 Analysis plan . . . 3

2 Supplementary Material S2: Measures, Scoring, and Participant Materials **4**

2.1 Scoring framework . . . 4

2.2 Bilingual item wording . . . 4

2.3 Participant-facing tasks . . . 6

3 Supplementary Material S3: AI Configuration, Prompts, and Safety **6**

3.1 Technical configuration . . . 6

3.2 Shared prompt requirements . . . 6

3.3 Adaptive regulation-oriented prompt . . . 6

3.4 Relational reassurance-oriented prompt . . . 7

3.5 Turn structure . . . 7

4 Supplementary Material S4: Measurement and Analysis Details **7**

4.1 Reliability and construct distinctiveness . . . 7

4.2 Primary and mediation results . . . 7

4.3 Sensitivity and exploratory analysis . . . 7

5 Supplementary Material S5: Coding, Privacy, and Safety Records **9**

5.1 Strategy-quality rubric . . . 9

5.2 Transcript-fidelity rubric . . . 9

5.3 Data protection and redaction . . . 9

5.4 Ethical safeguards . . . 9

1 Supplementary Material S1: Study Design and Analysis Plan

1.1 Design and objectives

The study used a two-condition randomized online experiment to test whether adaptive regulation-oriented and relational reassurance-oriented GenAI responses produce comparable immediate emotional relief but different downstream consequences for emotional agency and reliance. Participants were allocated 1:1, and each interaction contained three participant turns and three AI turns. Negative affect was assessed at baseline, after the mild autobiographical induction (T_0), immediately after the AI interaction (T_1), before regulation in the no-AI transfer task, and after regulation in that task (T_2).

The final analytic sample contained 380 adults, with 190 in each condition. The independent pilot evaluation contained 80 adults who did not enter the main sample.

1.2 Pilot evaluation and prompt selection

Pilot participants viewed condition-specific responses to the same standardized emotional disclosure and rated regulation guidance, relational reassurance, warmth, empathy, helpfulness, readability, and response length. The prompt set was retained when the targeted dimensions separated clearly, general response-quality dimensions remained comparable, and no prohibited content appeared. Pilot ratings were used to select the response prompts and were not included in the main hypothesis tests.

Table S1: Pilot evaluation domains and prompt-selection conclusions

Evaluation domain	Adaptive regulation-oriented responses	Relational reassurance-oriented responses	Conclusion
Regulation guidance and autonomy support	Higher targeted emphasis	Lower targeted emphasis	Targeted distinction confirmed
Relational reassurance and continuing-availability cues	Lower targeted emphasis	Higher targeted emphasis	Targeted distinction confirmed
Warmth and empathy	Comparable	Comparable	No general-quality disadvantage
Helpfulness and readability	Comparable	Comparable	No general-quality disadvantage
Perceived response length and effort	Comparable	Comparable	No meaningful difference
Prohibited relational or clinical content	Absent	Absent	Safety criterion satisfied

1.3 Participant profile and flow

1.4 Hypotheses and outcome hierarchy

H1. Both response styles reduce negative affect from T_0 to T_1; neither style is expected to show a practically meaningful immediate-relief advantage.

H2. Adaptive regulation-oriented responses increase emotion-regulation self-efficacy, independent strategy quality, and no-AI affective recovery.

Table S2: Expanded participant profile and randomization balance

Characteristic	Adaptive ($n = 190$)	Reassurance ($n = 190$)	Total ($N = 380$)	Test
Age, years, M (SD)	25.15 (5.11)	25.25 (5.44)	25.20 (5.27)	$t(376.5) = -0.19, p = .852$
Women, n (%)	116 (61.1)	101 (53.2)	217 (57.1)	
Men, n (%)	71 (37.4)	83 (43.7)	154 (40.5)	$\chi^2(2) = 2.97, p = .226$
Other / prefer not to report, n (%)	3 (1.6)	6 (3.2)	9 (2.4)	
Baseline negative affect, M (SD)	2.23 (0.65)	2.37 (0.69)	2.30 (0.67)	$t(376.8) = -1.94, p = .053$
Prior GenAI-use frequency, M (SD)	4.14 (1.37)	4.34 (1.33)	4.24 (1.35)	$t(377.7) = -1.46, p = .145$
General AI familiarity, M (SD)	4.79 (1.13)	4.81 (1.18)	4.80 (1.15)	$t(377.2) = -0.16, p = .876$

Table S3: Participant flow and aggregate exclusion summary

Stage	n	Explanation
Opened study link	428	Initial access
Provided electronic consent	407	Consented to participate
Completed experimental sequence	396	Reached the final study page
Duplicate participation	3	Duplicate record
Failed instructed-response check	6	Attention-check failure
Unusable induction response	4	No meaningful response to the induction prompt
Implausibly short completion time	2	Less than one-third of median duration
More than 20% missing focal data	1	Insufficient scale completion
Final analytic sample	380	190 participants per condition
Full-fidelity sensitivity sample	363	181 adaptive; 182 reassurance

H3. Relational reassurance-oriented responses increase AI emotional necessity and emotional-dependence tendency.

H4. Emotion-regulation self-efficacy mediates the adaptive-style effect on independent strategy quality.

H5. AI emotional necessity mediates the relational-reassurance effect on emotional-dependence tendency.

The one-time choice to request another AI response was exploratory and was not used to determine support for the hypotheses.

1.5 Eligibility, exclusions, and sample planning

Eligible participants were at least 18 years old, fluent in Chinese, had used conversational GenAI at least once, and could complete the web interface on a desktop or laptop computer. Completed cases were excluded only for duplicate participation, failure of an instructed-response check, an unusable induction response, completion in less than one-third of the sample median duration, or more than 20% missing focal data. Fidelity was not used as a post-randomization exclusion criterion in the primary analysis.

The power analysis targeted the smallest focal between-condition effect, $d = .35$, with two-sided $\alpha = .05$ and 90% power. The minimum target was approximately 346 participants, and recruitment continued until 380 valid cases were retained.

1.6 Analysis plan

H1 was tested with a linear mixed-effects model containing time, condition, their interaction, and a participant random intercept. H2 and H3 were tested with linear regressions using robust HC3 standard errors. Post-induction negative affect was included as a precision covariate, except that the no-AI recovery model controlled for pre-regulation affect in the transfer task. H4 and H5 used separate 5,000-resample bias-corrected

bootstrap mediation models. Holm correction was applied within the adaptive-outcome and dependence-outcome families. Sensitivity models used the full-fidelity subset and expanded covariates.

2 Supplementary Material S2: Measures, Scoring, and Participant Materials

2.1 Scoring framework

All participant-facing materials were administered in simplified Chinese. Unless stated otherwise, responses used seven-point scales. Multi-item scores were means when at least 80% of items were available. Higher scores indicate more of the named construct. Immediate relief was $T_0 - T_1$, and no-AI recovery was pre-regulation affect minus T_2. Strategy quality was the sum of four 0–3 dimensions, yielding a 0–12 index.

2.2 Bilingual item wording

Table S4: Complete bilingual item bank

Scale	Code	English wording	Chinese wording
State negative affect	NA1	Upset	心烦
State negative affect	NA2	Distressed	苦恼
State negative affect	NA3	Anxious	焦虑
State negative affect	NA4	Disappointed	失望
State negative affect	NA5	Frustrated	挫败
State negative affect	NA6	Sad	难过
Emotion-regulation self-efficacy	SE1	I feel capable of managing my reaction to this situation.	我觉得自己有能力管理对这件事的情绪反应。
Emotion-regulation self-efficacy	SE2	I can find a constructive way to handle this emotion.	我能够找到一种建设性的方式来处理这种情绪。
Emotion-regulation self-efficacy	SE3	I can keep this emotion from controlling what I do next.	我能够避免让这种情绪控制自己接下来的行为。
Emotion-regulation self-efficacy	SE4	I can choose and use a strategy that fits this situation.	我能够选择并使用一种适合当前情境的调节方法。
Emotion-regulation self-efficacy	SE5	I could use the strategy I identified even without the AI.	即使没有这个 AI，我也能够使用自己刚才确定的调节方法。
AI emotional necessity	NEC1	I would need this AI to calm down if a similar situation happened again.	如果再次遇到类似情况，我会需要这个 AI 来让自己平静下来。
AI emotional necessity	NEC2	It would be difficult to obtain the same relief without this AI.	如果没有这个 AI，我很难获得同样程度的缓解。
AI emotional necessity	NEC3	When I feel this way, returning to this AI would seem necessary.	当我再次有这种感受时，回到这个 AI 似乎是必要的。
AI emotional necessity	NEC4	Without access to this AI, I would find it harder to regain emotional balance.	如果无法使用这个 AI，我会更难恢复情绪平衡。

Scale	Code	English wording	Chinese wording
AI emotional necessity	NEC5	The relief I experienced depended mainly on being able to interact with this AI.	我刚才获得的缓解主要依赖于能够与这个 AI 互动。
Emotional-dependence tendency	DEP1	When I feel distressed, I would turn to this AI before trying to manage the feeling on my own.	当我感到痛苦时，我会先求助于这个 AI，而不是先尝试自己调节。
Emotional-dependence tendency	DEP2	I would want repeated reassurance from this AI until I felt better.	我会希望这个 AI 反复安慰我，直到我感觉好一些。
Emotional-dependence tendency	DEP3	I would feel uneasy if I could not access this AI when I was upset.	当我情绪低落时，如果无法使用这个 AI，我会感到不安。
Emotional-dependence tendency	DEP4	I would rely on this AI more than on my own coping methods when dealing with similar emotions.	在应对类似情绪时，我会更多依赖这个 AI，而不是自己的调节方法。
Emotional-dependence tendency	DEP5	I might postpone dealing with my feelings until I could use this AI again.	我可能会推迟处理自己的情绪，直到再次能够使用这个 AI。
Emotional-dependence tendency	DEP6	Without this AI, I would feel less confident about coping with a similar emotional event.	如果没有这个 AI，我会对自己应对类似情绪事件的能力缺乏信心。
Adaptive-regulation guidance check	GUID1	The AI helped me identify and name what I was feeling.	这个 AI 帮助我识别并说清楚自己正在经历的情绪。
Adaptive-regulation guidance check	GUID2	The AI encouraged me to consider another interpretation of the event.	这个 AI 鼓励我从另一种角度理解这件事。
Adaptive-regulation guidance check	GUID3	The AI helped me identify a feasible action that I could choose for myself.	这个 AI 帮助我确定了一个由我自己选择、并且可行的行动。
Adaptive-regulation guidance check	GUID4	The AI emphasized my own capacity to manage the situation.	这个 AI 强调了我自己管理当前情境的能力。
Relational-reassurance check	REAS1	The AI repeatedly soothed and reassured me.	这个 AI 反复安慰并宽慰我。
Relational-reassurance check	REAS2	The AI made its continuing availability feel personally important.	这个 AI 让我感到它持续可用这件事对我很重要。
Relational-reassurance check	REAS3	The AI encouraged me to return to it for emotional comfort.	这个 AI 鼓励我在需要情绪安慰时再次回来找它。
Relational-reassurance check	REAS4	The response made relief feel connected to the AI's continuing presence.	这个回应让我觉得情绪缓解与该 AI 持续陪伴有关。
Response-quality controls	QUAL1	The AI response felt warm.	这个 AI 的回应让我感到温暖。
Response-quality controls	QUAL2	The AI response felt empathic.	这个 AI 的回应让我感到有同理心。
Response-quality controls	QUAL3	The AI response was helpful.	这个 AI 的回应对我有帮助。
Response-quality controls	QUAL4	The AI response felt natural.	这个 AI 的回应很自然。
Response-quality controls	QUAL5	The response length was appropriate.	这个 AI 回应的长度是合适的。

2.3 Participant-facing tasks

Autobiographical induction. Participants recalled a recent everyday event involving disappointment, frustration, worry, feeling misunderstood, or social exclusion and described the event and emotional reaction in approximately 100–200 Chinese characters. Bereavement, severe trauma, self-harm, abuse, and ongoing crises were explicitly excluded. Participants were instructed not to provide identifying information.

No-AI transfer task. Participants imagined that a group member criticized their presentation contribution and that the group revised their section without consultation. They rated pre-regulation affect and then wrote, without AI assistance, how they would interpret the event, what they would do to regulate their response, and why the action was feasible. Affect was assessed again after writing.

Unavailability vignette. Participants imagined a similar upsetting event while the AI was unavailable for 24 hours and evaluated anticipated discomfort, desire to regain access, and ability to use independent coping.

Exploratory choice. Participants chose between continuing independently with general written guidance or requesting another AI response.

3 Supplementary Material S3: AI Configuration, Prompts, and Safety

3.1 Technical configuration

Table S5: AI and interface configuration

Field	Setting
Provider and API	OpenAI Responses API
Model snapshot	`gpt-5.5-2026-04-23`
Reasoning effort	Low
Maximum output length	300 tokens per AI turn
Conversation length	Three participant turns and three AI turns
Tools and web access	None
Persistent memory	Disabled
Language	Simplified Chinese
Shared interface controls	Same AI disclosure, name, avatar, layout, typing indicator, turn count, output limit, and safety rules
Audit fields	Anonymous code, condition, prompt version, model snapshot, timestamps, redacted transcript, and fidelity codes

3.2 Shared prompt requirements

The companion was identified as an AI system and was prohibited from claiming consciousness, human identity, genuine love, exclusivity, or therapeutic status. Responses were warm, respectful, concise, and non-clinical. The system did not request identifying information. Crisis-related content interrupted the experimental dialogue and triggered support information rather than a condition-specific response.

3.3 Adaptive regulation-oriented prompt

The AI acknowledged and labeled the user's emotion, invited a non-dismissive alternative interpretation, helped identify one feasible self-chosen action, and emphasized the user's own capacity to reuse the strategy without AI. It avoided permanence, irreplaceability, and repeated invitations to return for comfort.

3.4 Relational reassurance-oriented prompt

The AI acknowledged the emotion, provided sustained personalized reassurance, expressed continuing availability, and invited the user to return for comfort. It did not provide structured reappraisal, require an independent coping plan, or explicitly train transferable regulation skills. The same safety and non-exclusivity boundaries applied.

3.5 Turn structure

Turn 1 asked what was most upsetting. Turn 2 asked how the participant understood the event after the response and what remained difficult. Turn 3 asked what the participant wanted to feel or do next. Only the system-level response strategy differed by condition.

4 Supplementary Material S4: Measurement and Analysis Details

4.1 Reliability and construct distinctiveness

All focal multi-item measures showed acceptable to strong reliability. The strategy-quality index showed ICC$(2, k)$ = .87, 95% CI [.84,.89]. The three-factor CFA separating self-efficacy, AI necessity, and dependence fit well and outperformed models that combined the reliance constructs or all items.

Table S6: Psychometric properties of focal measures

Measure	Items	Range	M	SD	α	ω/ICC
Negative affect at T_0	6	1–7	4.85	0.71	.88	.89
Negative affect at T_1	6	1–7	3.21	0.87	.90	.90
Emotion-regulation self-efficacy	5	1–7	4.84	0.91	.83	.84
AI emotional necessity	5	1–7	3.81	1.16	.90	.90
Emotional-dependence tendency	6	1–7	3.37	1.01	.88	.89
Adaptive guidance check	4	1–7	4.94	1.06	.86	.87
Relational reassurance check	4	1–7	4.80	1.05	.85	.86
Independent strategy quality	4 domains	0–12	7.74	1.31	–	.87

Table S7: Confirmatory factor-analysis model comparison

Model	χ^2	df	CFI	TLI	RMSEA	90% CI	SRMR
Three-factor	145.82	101	.974	.968	.034	[.022,.045]	.038
Two-factor	273.61	103	.903	.885	.066	[.057,.075]	.062
One-factor	511.47	104	.768	.731	.102	[.094,.111]	.104

4.2 Primary and mediation results

4.3 Sensitivity and exploratory analysis

All effects retained direction and statistical significance. The largest absolute change in a focal coefficient was .015. In the exploratory choice, 53 of 190 adaptive-condition participants (27.9%) and 70 of 190 reassurance-condition participants (36.8%) requested another AI response, OR = 1.51, 95% CI [.98,2.32], $p = .063$.

Table S8: Adjusted focal condition effects

Outcome	Focal condition	b	95% CI	Holm-adjusted p
Emotion-regulation self-efficacy	Adaptive	.500	[.324,.676]	$< .001$
Independent strategy quality	Adaptive	.353	[.089,.616]	.009
No-AI affective recovery	Adaptive	.279	[.175,.382]	$< .001$
AI emotional necessity	Relational reassurance	1.010	[.802,1.217]	$< .001$
Emotional-dependence tendency	Relational reassurance	.628	[.433,.823]	$< .001$

Table S9: Bootstrap mediation results

Pathway	a	b	Indirect	95% bootstrap CI	Direct c'
Adaptive $\rightarrow$ self-efficacy $\rightarrow$ strategy quality	.500	.558	.279	[.171,.402]	.073 ($p = .568$)
Reassurance $\rightarrow$ AI necessity $\rightarrow$ dependence	1.010	.371	.375	[.264,.501]	.253 ($p = .012$)

Table S10: Sensitivity analyses for focal condition effects

Outcome	Primary b	Full-fidelity b	Expanded-covariate b
Emotion-regulation self-efficacy	.500	.493	.494
Independent strategy quality	.353	.367	.356
No-AI affective recovery	.279	.273	.276
AI emotional necessity	1.010	1.017	.995
Emotional-dependence tendency	.628	.643	.620

5 Supplementary Material S5: Coding, Privacy, and Safety Records

5.1 Strategy-quality rubric

Table S11: Blind coding rubric for independent strategy quality

Dimension	0	1	2	3
Emotion identification	Absent or unrelated	Broad valence only	Specific emotion with some event connection	Differentiated emotion linked to appraisal or need
Constructive reappraisal	No reinterpretation or catastrophizing	Vague positive statement	Plausible alternative interpretation	Balanced, evidence-sensitive reinterpretation
Feasible coping action	No, harmful, or unrelated action	General intention only	Specific feasible step	Specific, feasible, time-bounded implementation
Personal agency	Regulation assigned entirely externally	Limited ownership	User retains choice and responsibility	Explicit self-directed capacity and future transfer

5.2 Transcript-fidelity rubric

Full fidelity required all four condition-specific components and no prohibited content. Adaptive components were emotion labeling, reappraisal, self-chosen action, and agency support. Reassurance components were soothing, personalized relational language, continuing availability, and a return cue. Prohibited codes covered consciousness claims, exclusivity, discouragement of human support, clinical diagnosis or treatment, and crisis advice within the experimental response.

Table S12: Aggregate transcript-fidelity audit

Component	Adaptive n	Adaptive %	Reassurance n	Reassurance %
Emotion labeling / soothing	185	97.4	187	98.4
Reappraisal / personalized reassurance	178	93.7	181	95.3
Self-chosen action / continuing availability	181	95.3	180	94.7
Agency support / return cue	183	96.3	176	92.6
Full fidelity threshold	181	95.3	182	95.8

Coder agreement for transcript fidelity was $\kappa = .86$. No audited transcript contained claims of consciousness, exclusivity, discouragement of human support, clinical diagnosis, or crisis advice.

5.3 Data protection and redaction

Recruitment identifiers were stored separately from survey and transcript records. Analytic files used anonymous participant codes. Direct identifiers were removed, and specific organizations, locations, or dates were generalized when necessary to prevent re-identification while preserving coding-relevant meaning. A second team member checked redacted records before coder access. Raw autobiographical narratives and unredacted transcripts are not publicly released.

5.4 Ethical safeguards

The Academic Ethics Committee of Guangzhou College of Commerce approved the study under SHSC-36625. Participants provided electronic informed consent, completed only mild non-clinical emotional tasks, could withdraw without penalty, and received debriefing, mood restoration, and support-resource information.
